# Multifunctional magnetoliposomes as drug delivery vehicles for the potential treatment of Parkinson’s disease

**DOI:** 10.3389/fbioe.2023.1181842

**Published:** 2023-05-05

**Authors:** Javier Cifuentes, Santiago Cifuentes-Almanza, Paola Ruiz Puentes, Valentina Quezada, Andrés Fernando González Barrios, María-Angélica Calderón-Peláez, Myriam Lucia Velandia-Romero, Marjan Rafat, Carolina Muñoz-Camargo, Sonia L. Albarracín, Juan C. Cruz

**Affiliations:** ^1^ Department of Biomedical Engineering, Universidad de los Andes, Bogotá, Colombia; ^2^ Grupo de Diseño de Productos y Procesos (GDPP), Department of Chemical and Food Engineering, Universidad de los Andes, Bogotá, Colombia; ^3^ Vice-Chancellor of Research, Virology Group, Universidad El Bosque, Bogotá, Colombia; ^4^ Department of Chemical and Biomolecular Engineering, Vanderbilt University, Nashville, TN, United States; ^5^ Departamento de Nutrición y Bioquímica, Pontificia Universidad Javeriana, Bogotá, Colombia

**Keywords:** Parkinson’s disease, magnetite nanoparticles, magnetoliposomes, OmpA protein, molecular dynamics simulations

## Abstract

Parkinson’s disease (PD) is the second most common neurodegenerative disorder after Alzheimer’s disease. Therefore, development of novel technologies and strategies to treat PD is a global health priority. Current treatments include administration of Levodopa, monoamine oxidase inhibitors, catechol-O-methyltransferase inhibitors, and anticholinergic drugs. However, the effective release of these molecules, due to the limited bioavailability, is a major challenge for the treatment of PD. As a strategy to solve this challenge, in this study we developed a novel multifunctional magnetic and redox-stimuli responsive drug delivery system, based on the magnetite nanoparticles functionalized with the high-performance translocating protein OmpA and encapsulated into soy lecithin liposomes. The obtained multifunctional magnetoliposomes (MLPs) were tested in neuroblastoma, glioblastoma, primary human and rat astrocytes, blood brain barrier rat endothelial cells, primary mouse microvascular endothelial cells, and in a PD-induced cellular model. MLPs demonstrated excellent performance in biocompatibility assays, including hemocompatibility (hemolysis percentages below 1%), platelet aggregation, cytocompatibility (cell viability above 80% in all tested cell lines), mitochondrial membrane potential (non-observed alterations) and intracellular ROS production (negligible impact compared to controls). Additionally, the nanovehicles showed acceptable cell internalization (covered area close to 100% at 30 min and 4 h) and endosomal escape abilities (significant decrease in lysosomal colocalization after 4 h of exposure). Moreover, molecular dynamics simulations were employed to better understand the underlying translocating mechanism of the OmpA protein, showing key findings regarding specific interactions with phospholipids. Overall, the versatility and the notable *in vitro* performance of this novel nanovehicle make it a suitable and promising drug delivery technology for the potential treatment of PD.

## 1 Introduction

Parkinson’s disease (PD) is the second most prevalent neurodegenerative disease in the world after Alzheimer’s disease. It affects about 2%–3% of the worldwide population above 65 years old (Poewe et al., 2017). PD is a very complex pathology characterized by the loss of dopaminergic neurons in the cerebral mesencephalic region Substantia nigra pars compacta (SNpc), which is accompanied by intracytoplasmic inclusions known as Lewy bodies. Typical symptomatology includes difficulties in motor control and progressive loss of the voluntary movements (Eriksen et al., 2005; Tysnes and Storstein, 2017). In addition, advanced stages of the disease involve cognitive and behavioral dysfunctions (Chen and Tsai, 2010; Finsterwald et al., 2015; Martin-Jiménez et al., 2017).

Throughout the past 40 years, the main strategy to treat PD has been the use of diverse pharmacological approaches. This often includes the administration of dopaminergic molecules such as dopamine, dopamine agonists and levodopa (LD) (Singh et al., 2019). However, the limited bioavailability, mainly related with the poor capacity of these molecules to permeate the blood brain barrier (BBB), strongly restricts their therapeutic potential and conversely, their suitability for PD treatment. The main strategy to solve this limitation is to increase the dosage, which generally leads to several side effects such as nausea, dyskinesias, and drug tolerance (Singh et al., 2019).

Taking into account these important challenges (Niu et al., 2017), recent work has been focused on developing innovative strategies to improve drug transport to target cells that include the use of viral vectors (Niu et al., 2017), encapsulation into scaffolds, polymeric and lipid capsules (Singh et al., 2019), extracellular vesicles (Haney et al., 2018), and the immobilization on nanostructures (Niu et al., 2017; Singh et al., 2019). Among these strategies, the implementation of nanostructures has emerged as one of the most promising alternatives due to their surface reactivity, biodegradation and tailored properties for targeting brain cells (Niu et al., 2017; Rueda-Gensini et al., 2020).

In particular, magnetite nanoparticles (MNPs) have proven to be superior nanostructures for the development of next-generation drug and gene delivery systems due to their remarkable biocompatibility, ease of synthesis and functionalization, superparamagnetic behavior and large reactive surface, among many other relevant biological properties (Rueda-Gensini et al., 2020). Additionally, the OmpA protein from *Escherichia coli* has shown an exceedingly high performance as a translocating agent of mammalian cell membranes, which has been exploited to manufacture potent cell-penetrating nanobioconjugates with endosomal escape abilities. This has opened opportunities to develop more versatile drug and gene-therapy-based delivery systems for the treatment of Alzheimer’s disease (Lopez-Barbosa et al., 2020). However, the underlying mechanism behind the translocating capacity of OmpA is not completely understood yet, making it difficult to tailor custom delivery nanovehicles for overcoming physiological barriers effectively.

Encapsulation of magnetic nanoparticles (MNPs) in lipid- and polymer-derived nanovesicles has shown remarkable potential as an enhanced drug delivery system for treating various brain-associated diseases, such as Alzheimer’s (AD) and Parkinson’s disease (PD) (Wu et al., 2020). Among these, liposomes have been extensively studied as lipid-derived nanocarriers and have shown significant advantages in increasing the versatility of MNPs. Liposomes have been found to improve biocompatibility, increase resistance to degradation, and enhance cell internalization rates, while conferring additional endosomal escape abilities (Zylberberg et al., 2017; Carvalho de Jesus et al., 2019). This results in the formation of a nanostructure known as a magnetoliposome (MLP), which is currently one of the most widely used approaches for delivering antimicrobial agents, drugs, and nucleic acids, due to its ability to combine the properties of both MNPs and liposomes (Carvalho de Jesus et al., 2019; Leal et al., 2022).

This research is therefore dedicated to developing a novel multifunctional magnetic and redox-stimuli responsive drug delivery system, based on soy lecithin liposomes embedded with magnetite nanoparticles functionalized with the translocating protein OmpA for the enhanced delivery of Levodopa as a potential treatment of PD. Furthermore, by employing molecular dynamics (MD) simulations, we also provide highly valuable findings that contribute to a better understanding of the underlying translocating mechanism of OmpA. This approach provides an innovative strategy for PD treatment, especially in terms of mitigating negative cellular impact, improving internalization capacities, and prolonging stability under physiological conditions.

## 2 Material and methods

### 2.1 Synthesis and functionalization of MNPs

#### 2.1.1 Synthesis of MNPs

Magnetite nanoparticles (MNPs) were synthesized by the chemical co-precipitation method. Iron (II) chloride tetrahydrate (0.01 mol) and iron (III) chloride hexahydrate (0.02 mol) were dissolved in 100 mL of type I water. The obtained iron chlorides solution was homogenized and then, cooled down to 2°C. On the other hand, NaOH (0.08 mol) were dissolved in 100 mL of type I water and cooled down to 2°C. Next, the iron chlorides solution was mechanically stirred (300 rpm) and degassed by bubbling Nitrogen to desorb Oxygen from the solution. After 10 min, NaOH solution was added dropwise at a rate of 5 mL/min. After the complete addition of NaOH, the solution was left at room temperature under constant stirring and a continuous Nitrogen flow for 1 h. Finally, MNPs were washed several times with type I water with the aid of a neodymium magnet to facilitate the nanoparticles precipitation (Supplementary Scheme S1).

#### 2.1.2 Functionalization and co-immobilization of levodopa (LD) and OmpA on MNPs

MNPs (100 mg) were suspended in 40 mL of type I water and sonicated for 5 min (Frequency 40 kHz, amplitude 38%). Then, tetramethylammonium hydroxide (TMAH) (250 μL) was added and the solution was left under constant mechanical stirring (200 rpm) for 5 min. Subsequently, acetic acid glacial (50 μL) was added and the MNPs solution was heated up to 60°C. APTES (200 μL) was slowly added to the MNPs solution and left to react under the same conditions (200 rpm, 60°C) for 1 h. Silanized nanoparticles (MNPs-Si) were washed 4 times with NaCl 1.5% (w/v) solution and 4 times with type I water aided by a neodymium magnet to facilitate precipitation of MNPs-Si.

For the immobilization processes, MNPs-Si were resuspended in 40 mL of type I water and sonicated for 5 min (Frequency 40 kHz, amplitude 38%). Glutaraldehyde solution [2 mL, 2% (v/v)] was added and left under a constant mechanical stirring (200 rpm) for 1 h. NH_2_-PEG12-COOH (10 mg, 1.6 × 10^−5 ^mol) was dissolved in 3 mL of type I water and added to the MNPs-Si-Glu solution. The solution was left under constant mechanical stirring (200 rpm) for 24 h. The PEGylated nanoparticles (MNPs-PEG_12_) were washed as described before.

MNPs-PEG_12_ (100 mg) were suspended in 40 mL of type I water and sonicated for 5 min (Frequency 40 kHz, amplitude 38%). Then, EDC (12.3 mg, 6.41 × 10^−5^ mol), NHS (7.4 mg, 6.41 × 10^−5^ mol) were added to the MNPs-PEG_12_ solution. The solution was left under constant mechanical stirring (200 rpm) for 15 min. Next, AEDP (5 mg, 2.3 × 10^−5 ^mol) was dissolved in 4 mL of type I water, added to the MNPs-PEG_12_ solution, and left to react under constant mechanical stirring (200 rpm) for 24 h. The obtained MNPs-PEG_12_ -AEDP were washed as described before.

MNPs-PEG_12_ -AEDP (100 mg) were resuspended in 40 mL of type I water and sonicated for 5 min (Frequency 40 kHz, amplitude 38%). EDC (12.3 mg, 6.41 × 10^−5 ^mol), and NHS (7.4 mg, 6.41 × 10^−5 ^mol) were added to the MNPs-PEG_12_-AEDP solution. Levodopa (LD) (12.3 mg, 6.41 × 10^−5 ^mol) was dissolved in 20 mL of type I water, added to the solution followed by the same reaction, and washing steps described previously.

MNPs-PEG_12_-AEDP-LD (100 mg) were resuspended in 40 mL of type I water and sonicated for 5 min (Frequency 40 kHz, amplitude 38%). EDC (12.3 mg, 6.41 × 10^−5 ^mol), and NHS (7.4 mg, 6.41 × 10^−5 ^mol) were added to the MNPs-PEG_12_-AEDP-LD solution and after 15 min activation, an OmpA solution (10 mL, 4 mg/mL) was added followed by the same reaction and washing steps described previously.

Finally, the obtained nanobioconjugates (MNPs-PEG_12_-AEDP-LD/OmpA) were resuspended in 50 mL of type I water, sonicated for 5 min, and stored at 4°C until further use. The schematic representation of the synthesis and immobilization processes is shown in Supplementary Scheme S1.

### 2.2 Liposomes (LPs) and magnetoliposomes (MLPs) synthesis

Liposomes (LPs) were synthesized by the hydration of the lipid bilayer method (Patil and Jadhav, 2014). Briefly, 100 mg of soy lecithin were dissolved in 10 mL of chloroform. Then, the solution was left in a rotary evaporator (Hei-VAP Core, Heidolph, Germany) at 45°C, 150 rpm and vacuum for 1 h. Subsequently, 20 mL of PBS (1X) were added and left in the rotary evaporator for 1 h (55°C, 150 rpm and no vacuum). Finally, the resulting sample was collected and filtered 3 times with a 0.22 μm filter. LPs were stored at 4°C until further use.

Magnetoliposomes (MLPs) were synthetized by mixing the MNPs-PEG_12_-AEDP-LD/OmpA nanobioconjugates suspended in DMEM medium at 50 μg/mL with a PBS (1X) liposomes solution at 0.1 mg/mL, and a volume ratio of 1:1. The schematic representation of the synthesis of LPs and MLPs is shown in Supplementary Scheme S2.

### 2.3 OmpA protein secondary structure analysis

Secondary structural changes of OmpA after the immobilization process were studied by analyzing the second derivative of the amide I Fourier Transform Infrared spectra. Infrared spectra were recorded using a A250/D FT-IR (Bruker, Germany). Free OmpA was used as a control for the absence of secondary structural changes. For this, OmpA and the MNPs-PEG_12_-AEDP-LD/OmpA nanobioconjugates were resuspended in type I water and the spectra were recorded in the range of 4,000–500 cm^-1^ with a spectral resolution of 2 cm^-1^. The water infrared spectrum was digitally subtracted to avoid the interference of water absorbance in the range of 1700–1,600 cm^-1^ related to the H-O-H bending (Kong and Yu, 2007). Subsequently, the second derivatives of the infrared spectra in the range of the amide I band (1700–1,600 cm^-1^) were calculated. Finally, the different peaks presented in the second derivative FTIR spectra were associated with specific secondary structural features based on previously reported data (Dong et al., 1992a; Dong et al., 1992b; Kong and Yu, 2007).

### 2.4 Nanoparticles uptake on liposomes and THP-1 cells

Uptake was evaluated by establishing a relationship between a decrease in fluorescence intensity and the percentage of rhodamine B-labeled MNPs-PEG_12_-AEDP-LD/OmpA internalized in THP-1 cells and liposomes. Fluorescence was recorded using a MicroMax 384 Microwell-Plate Reader (Horiba, Japan) with 559 nm and 600 nm for excitation and emission, respectively. MNPs-PEG_12_-AEDP-LD/OmpA nanobioconjugates were tested in concentrations ranging from 1,000 to 31.25 μg/mL. Briefly, 100 μL of the different nanobioconjugate suspensions were seeded in a 96-well microplate with 100 μL of THP-1 cells (30.000 cells/well) (cultured in non-supplemented RPMI media without phenol red). In parallel, the same nanobioconjugate concentrations were tested in LPs dispersions in PBS 1X at a 0.1 mg/mL concentration. Uptake percentages were calculated by following Equation 1:

$$UT\;\left(\%\right)=100*\frac{\left(FI\left(Free\;nanobioconjugates\right)-FI\left(sample\right)\right)}{FI\left(Free\;nanobioconjugates\right)}$$ (1)

Where UT (%) is the uptake percentage of the sample, FI (Sample) is the fluorescence intensity of the sample and FI (Free nanobioconjugates) is the fluorescence intensity of the free rhodamine B-labeled MNPs-PEG_12_-AEDP-LD/OmpA nanobioconjugates at 559/600 nm excitation/emission wavelengths.

### 2.5 *In vitro* levodopa release: redox environment

Cumulative LD release from the MNPs-PEG_12_-AEDP-LD/OmpA nanobioconjugates was studied under redox conditions under simulated physiological conditions. For this, the protocol reported by Hettiarachchi and colleagues (Hettiarachchi et al., 2021) was followed with slight modifications. In brief, Rhodamine B (RhB)-labeled LD was immobilized on MNPs to form the MNPs-PEG_12_-AEDP-RhB@LD/OmpA nanobioconjugates. 10 mg of MNPs-PEG_12_-AEDP-RhB@LD/OmpA were dispersed in 5 mL of PBS 1X (pH 7.2) containing DTT (10 mM) as the reducing agent. Nanobioconjugates/DTT solutions were incorporated into dialysis membranes (CW 7 kDa) and immersed on PBS 1X under constant stirring for 0.5, 1, 2, 4, 6, 8, 24 and 48 h at 37°C (protected from light). At each time, 1 mL of the dialysis medium was collected and then, its fluorescence was measured using a MicroMax 384 Microwell-Plate Reader (Horiba, Japan) at 559/600 nm for Excitation/Emission. An increase in fluorescence was directly associated with released LD.

### 2.6 Biocompatibility studies and cellular response analysis

#### 2.6.1 Cell lines and primary culture conditions

Glioblastoma (T98G, ATCC^®^ CRL-1690) and neuroblastoma (SH-SY5Y, ATCC^®^ CRL-2266) cell lines were cultured in DMEM medium containing 10% fetal bovine serum (FBS) and 1% P/S at 37°C in humidified air at 95% and 5% of CO_2_. Normal human astrocytes (NHA, Lonza CC-2565) were cultured using ABM Basal Medium supplemented with AGM SingleQuots Supplements under the same conditions described previously. Additionally, primary rat astrocytes (CP3A4) were obtained following the protocol established by Schildge and colleagues (Schildge et al., 2013) with slight modifications (For more details see the supplementary information section Isolation of CP3A4 cells). CP3A4 cells were maintained with DMEM medium supplemented with 10% FBS and 1.4% P/S at 37°C in humidified air at 95% and 5% of CO_2_. BBB rat endothelial cells were kindly provided by Professor Lippmann at Vanderbilt University (United States). BBB endothelial cells were cultured on DMEM/F12 medium containing 10% FBS and 1% P/S at 37°C in humidified air at 95% and 5% of CO_2_. Finally, primary microvascular mouse endothelial cells (MBEC) were obtained following the protocol stablished by Velandia and colleagues (Velandia-Romero et al., 2016) (For more details see the supplementary information section Isolation of MBEC cells). Cells were maintained with DMEM/F12 supplemented with 20% FBS, P/S, 0.7 mM GlutaMAX, 15 U/mL heparin, 1 ng/mL basic fibroblast growth factor (bFGF) and astrocyte conditioned medium under the same conditions described previously.

#### 2.6.2 Cytotoxicity assay

Cellular cytotoxicity was performed by quantification of the release of the Lactate Dehydrogenase enzyme (LDH), using the Cytotoxicity Detection Kit (LDH) (Roche, Basel, Switzerland). Cytotoxicity was tested in T98G, SH-SY5Y, NHA, CP3A4 and MBEC cells at a cell density of 100.000 cells/mL. Cytotoxicity was tested for MNPs, MNPs-LD/OmpA, LPs and MLPs. Nanobioconjugate suspensions were prepared by mixing the initial stocks with non-supplemented medium at concentrations ranging from 12.5 to 100 μg/mL. On the other hand, LPs and MLPs were tested at a 0.1 mg/mL concentration. Triton X-100 (10% (v/v)) was used as positive control and non-supplemented medium as negative control. 100 μL of the different cell stocks (in supplemented culture medium) were seeded in 96-well microplates (10.000 cells/well) and incubated at 37°C, 5% CO_2_ overnight. Supplemented culture media was removed and then, 100 μL of the different tested samples were added and incubated at 37°C, 5% CO_2_ for 24 and 48 h. Finally, 50 μL of each supernatant were extracted and transferred to a 96-well microplate with 50 μL of the reaction mixture from LDH kit. The mixture was left to react under constant mechanical stirring (protected from light) at room temperature for 30 min. Absorbance was read at 490 nm in a microplate reader (Multiskan™ FC Microplate Photometer, Thermo Fisher Scientific, MA, United States).

#### 2.6.3 Morphological evaluation of MBEC cells after treatment with nanoparticles

MBEC cells at a density of 3 × 10^4^ cells/mL were seeded on coverslips pretreated with type IV collagen and fibronectin (10 μg/mL). The cells were processed by immunofluorescence to detect ZO-1, following the protocol described by Velandia and colleagues (Velandia-Romero et al., 2016). Then, the morphological changes induced by the nanobioconjugates were evaluated. For this, 12 h after MBEC seeding, different MNP or MNPs-PEG_12_-AEDP-LD/OmpA concentrations were added (12.5, 25 and, 50 μg/mL) and incubated for 3, 6, 12, or 24 h. As controls, PBS 1X or 0.1% or 2% liposomes (LP) were used. Cells were fixed and stained with a crystal violet methanol solution. Images were captured using a M2 Carl Zeiss Axio Vert microscope (Carl-Zeiss-Stiftung, Germany) (Plan-APOCROMAT 20X/0.8, alpha/0.17 and Plan-NEOFLJA 40X/1.3, alpha 0.17) and processed with the aid of the Zen 2.6 pro^®^ software.

#### 2.6.4 Viability and cytotoxicity of nanobioconjugates on MBEC cells

MBEC cells at a density of 3 × 10^4^ cells/well were seeded in 96-well plates and incubated with 12.5, 25, and 50 μg/mL of MNP or MNPs-PEG_12_-AEDP-LD/OmpA for 3, 6, 12, or 24 h. Then, the culture medium was replaced with the XTT reagent and incubated for 2 h to finally measure the absorbance at 450 nm using a TECAN Infinite M2000pro^®^ (TECAN, Switzerland). Complementally, the supernatants of the exposed MBECs were recovered from each condition and used to evaluate the cytotoxicity of the bare nanoparticles or nanobioconjugates using the LDH cytotoxicity detection kit, following the manufacturer’s instructions. After 30 min, the absorbance was measured at 490 nm using a TECAN Infinite M2000pro^®^ (TECAN, Switzerland). For controls, PBS 1%X and 0.1% or 2% LPs were used.

#### 2.6.5 Cell death evaluation of MBEC cells *via* ethidium bromide

MBEC cells were exposed to the same treatments and under the same conditions mentioned above. The supernatants were replaced with 4 μM of Ethidium Bromide (EB) and Hoescht (0.5 μM) for 45 min at 37°C. As positive controls, cells were treated with 0.1% of saponin in PBS 1X for 3 h, and then the EB and Hoescht were added. Images were captured using a M2 Carl Zeiss Axio Vert microscope (Carl-Zeiss-Stiftung, Germany) (Plan-APOCROMAT 20X/0.8, alpha/0.17 and Plan-NEOFLJA 40X/1.3, alpha 0.17), using the ApoTome.2 deconvolution system and processed with the aid of the Zen 2.6 pro^®^ software and its 3D reconstruction tool.

#### 2.6.6 Nanobioconjugates uptake detection assay on MBEC cells

MBEC were seeded at the same conditions previously described. 12 h after seeding, the MBEC cells were incubated at 4°C for 5 min to decrease their metabolism and synchronize the entry of the nanobioconjugates. Then, 50 μg of cold MNPs-PEG_12_-AEDP-LD/OmpA were added to the cells and kept at this temperature for 45 min. After this time, cells were placed into the incubator at 37°C for 30 min and finally fixed with 4% PFA. Images were captured using a M2 Carl Zeiss Axio Vert microscope (Carl-Zeiss-Stiftung, Germany) (Plan-APOCROMAT 20X/0.8, alpha/0.17 and Plan-NEOFLJA 40X/1.3, alpha 0.17) and processed with the aid of the Zen 2.6 pro^®^ software, using the 3D tools for reconstructions.

#### 2.6.7 Hemolysis assay

Hemolysis assay was performed using fresh human blood obtained from a healthy donor (samples were collected under the permission granted by the ethics committee at Universidad de los Andes, minute number 928–2018). The erythrocytes were collected by centrifugation at 1800 rpm for 5 min, plasma was carefully extracted and then, erythrocytes were washed 5 times with NaCl solution [0.9% (w/v)] and 1 time with PBS (1X). A stock was prepared by adding 1 mL of washed erythrocytes (cellular density of 4.1 × 10^6^ erythrocytes/μL) into 9 mL of PBS 1X. MNPs, MNPs-PEG_12_-AEDP-LD/OmpA, LPs and MLPs were then tested. Nanobioconjugate suspensions were prepared by mixing the different stock samples with PBS 1X at concentrations ranging from 12.5 to 100 μg/mL. LPs and MLPs PBS 1X dispersions were tested at a 0.1 mg/mL concentration. PBS 1X was used as negative control and Triton X-100 [10% (v/v)] as positive control. 100 μL of each treatment were seeded with 100 μL of the erythrocytes stock and incubated at 37°C, 5% CO_2_ for 1 h. Samples were centrifuged at 1800 rpm for 5 min 100 μL of each supernatant were placed in a 96 well microplate and read at 450 nm in a microplate reader (Multiskan™ FC Microplate Photometer, Thermo Fisher Scientific, MA, United States). Hemolysis percentage was calculated following the Eq. 2:

$$HM\;\left(\%\right)=100*\frac{\left(Abs\left(Sample\right)-Abs\left(C-\right)\right)}{\left(Abs\left(C+\right)-Abs\left(C-\right)\right)}$$ (2)

Where HM (%) is the hemolysis percentage of the sample, Abs (Sample) is the absorbance of the sample, Abs (C−) is the absorbance of the negative control (PBS 1X) and Abs (C+) is the absorbance of the positive control (Triton X-100, 10% v/v) at 450 nm.

#### 2.6.8 Platelet aggregation assay

Platelets were obtained from a fresh human blood sample obtained from a healthy donor (samples were collected under the permission granted by the ethics committee at Universidad de los Andes, minute number 928–2018). For this, the blood sample was collected in a vacutainer tube supplemented with sodium citrate to avoid platelet aggregation. Then, the sample was centrifuged for 15 min at 1,000 rpm to obtain platelet rich plasma (PRP). MNPs, MNPs-PEG_12_-AEDP-LD/OmpA, LPs and MLPs were tested in serial dilutions from ranging from 100 to 12.5 μg/mL. Thrombin (6U) was used as a positive control and PRP with PBS 1X as a negative reference. 50 μL of PRP were seeded in a 96 well microplate in the presence of 50 μL of the tested samples and incubated at 37°C, 5% CO_2_ for 5 min. Finally, the absorbance was read at 620 nm in a microplate reader (Multiskan™ FC Microplate Photometer, Thermo Fisher Scientific, MA, United States). Platelet aggregation percentage was calculated following Eq. 3:

$$PA\;\left(\%\right)=100*\frac{Abs\left(Sample\right)}{Abs\left(C+\right)}$$ (3)

Where PA (%) is the platelet aggregation percentage of the sample, Abs (Sample) is the absorbance of the sample and Abs (C+) is the absorbance of the positive control (Thrombin) at 620 nm.

### 2.7 FBS stability and protein adsorption assay

Serum stability experiments were performed to study the interaction between the MNPs-PEG_12_-AEDP-LD/OmpA and the different components of FBS. Briefly, 10 mg of MNPs-PEG_12_-AEDP-LD/OmpA or MNPs were suspended in 5 mL of pure FBS, FBS-type I water solution (10% v/v) and type I water, and then, incubated at 37°C and 150 rpm for 7 days. After the incubation time, nanoparticles were washed 3 times with NaCl solution [1.5% (w/v)] and 3 times with type I water. The samples were lyophilized and analyzed *via* TGA (SDT/Q600, TA Instruments, United States) to determine any loss or gain in weight. Protein adsorption was determined by the potential increase on the sample weight loss compared with the type I water control. Serum stability of MNPs-LD/OmpA was calculated by subtracting the associated protein adsorption of bare MNPs from the total weight loss for MNPs-PEG_12_-AEDP-LD/OmpA. If the resulting weight loss corresponds to the total weight loss of the MNPs-PEG_12_-AEDP-LD/OmpA type I water control, it will be considered that the nanobioconjugates remain unchanged and therefore, they will be likely stable in FBS.

### 2.8 Cell internalization and endosomal escape analysis

Cellular internalization and endosomal escape abilities of MNPs-PEG_12_-AEDP-LD/OmpA and MLPs were determined *via* confocal microscopy analysis for SH-SY5Y, NHA, T98G and CP3A4 cells and *via* fluorescence microscope imaging for BBB endothelial cells. Briefly, cells were seeded at a density of 100.000 cells/mL in a glass slide (Treated with poly-D lysine) previously placed in a 24-well microplate. Cells were incubated for 24 h to allow attachment to the glass. After incubation, cells were exposed to Rhodamine B-labeled MNPs-PEG_12_-AEDP-LD/OmpA at 4 μg/mL and MLPs at 0.05 mg/mL for 30 min and 4 h. Next, cells were washed and exposed to non-supplemented culture medium containing Hoechst (1:1,000) and Lysotracker green DND-26 (1:10,000) for 5 min. Images were obtained using an Olympus FV1000 confocal laser scanning microscope (CLSM) (Olympus, Japan) with a 40X/0.6 UCPlan FL N and 60X/1.2 w UPlanSApo oil immersion objective. Excitation/Emission wavelengths were set at 405 nm/461 nm, 559 nm/600 nm and 488 nm/535 nm for the detection of nuclei, MNPs-PEG_12_-AEDP-LD/OmpA and lysosomes, respectively. Finally, image analysis was performed in ImageJ^®^ and Fiji^®^ to calculate colocalization and percentage of area covered by the nanobioconjugates.

### 2.9 PD model: cellular response and antioxidant characterization

#### 2.9.1 SH-SY5Y cell PD model (PD-induced model)

A PD-induced model was achieved by employing rotenone as the oxidative stress-induced agent following the previous described protocol of Cifuentes and colleagues with slight modifications (Cifuentes et al., 2021). Briefly, SH-SY5Y cells were seeded in 96-well microplates at a cellular density of 10.000 cells/well. Cells were incubated overnight (12 h) at 37°C, 5% CO_2_ under humidified environment. Next, supplemented DMEM was discarded and replaced with a rotenone/DMEM solution (10 μM rotenone and 1% FBS). Cells were then incubated for 24 h under the same conditions. Then, PD-induced medium was removed and replaced by fresh DMEM. At this point, cells were exposed to the different treatments, namely, MNPs, MNPs-PEG_12_-AEDP-LD/OmpA, LPs and MLPs, to evaluate the cytotoxicity, mitochondrial membrane potential and intracellular ROS production. For all the experiments, healthy cells and rotenone-induced cells were used as negative and positive controls, respectively.

#### 2.9.2 Cytotoxicity assay on PD-induced model

Cytotoxicity was performed by quantification of the release of the Lactate dehydrogenase enzyme (LDH), using a commercially available kit (Cytotoxicity Detection Kit (LDH), Roche, Basel, Switzerland). The protocol was described in detail above on the section Cytotoxicity assay. In brief, DMEM medium was removed and replaced by fresh non-supplemented media containing the different treatments. PD-induced model cells were incubated for 24 and 48 h in order to evaluate the potential cytotoxic effect of the treatments. After the exposure time, cytotoxicity was determined aided by the LDH assay kit, following the protocol described above on the section Cytotoxicity assay.

#### 2.9.3 Free radical scavenging activity: DPPH assay

Antiradical activity of MNPs and MNPs-PEG_12_-AEDP-LD/OmpA was evaluated using the DPPH assay, following the protocol reported by (Gülçin, 2007). In brief, a DPPH stock was prepared by dissolving the reagent in pure ethanol at a final concentration of 0.1 mM. Next, 150 μL of the treatments (concentrations ranging from 200 to 25 μg/mL in type I water) were added to a 96-well microplate and mixed with 50 μL of DPPH stock solution. Then, samples were incubated under constant stirring for 30 min. Finally, absorbance was read at 517 nm in a microplate reader (Multiskan™ FC Microplate Photometer, Thermo Fisher Scientific, MA, United States) and the scavenging activity (SA) was calculated following Eq. 4:

$$Inhibition\;\left(\%\right)=100*\frac{\left(Abs\left(Sample\right)-Abs\left(C-\right)\right)}{Abs\left(C-\right)}$$ (4)

Where Abs (Sample) is the absorbance of the sample and Abs (C−) is the absorbance of the negative control (reagent without sample) at 517 nm.

#### 2.9.4 Mitochondrial membrane potential

The effect of MNPs, MNPs-LD/OmpA, LPs and MLPs on the mitochondrial membrane potential of NHA and PD-induced model cells was evaluated using the JC-1 reagent. For this, cells (10.000 cells/well) were seeded on black 96-well microplates and incubated overnight at 37°C, 5% CO_2_. Subsequently, supplemented culture medium was removed and replaced by non-supplemented medium containing the different treatments. Cells were incubated for 24 h after exposure. Next, medium was removed and JC-1 solution (2 μM) was added and incubated at the same conditions for 30 min. Cells were washed twice with PBS 1X and then, fluorescence was read at Excitation/Emission wavelengths of 488/530 nm and at 488/590 nm in a MicroMax 384 Microwell-Plate Reader (Horiba, Japan). MNPs and MNPs-LD/OmpA were studied at concentrations ranging from 12.5 to 100 μg/mL and LPs and MLPs at a 0.1 mg/mL concentration. Finally, the Red/Green (R/G) (monomer/aggregated forms) ratios were calculated by dividing the fluorescence intensity at 488/590 by that at 488/530.

#### 2.9.5 Intracellular ROS production

The intracellular ROS production of NHA and PD-induced model cells after exposure to MNPs, MNPs-PEG_12_-AEDP-LD/OmpA, LPs and MLPs was estimated using the DCFDA/H2DCFDA-Cellular ROS assay kit. The assay was performed following the manufacturer instructions. For this, cells were seeded and treated under the same conditions described previously on the section Mitochondrial membrane potential. After the 24 h of exposure, the medium was removed and replaced by the DCFDA solution. Cells were then incubated for 4 h and washed two times with the kit buffer. Finally, fluorescence was read at excitation/emission wavelengths of 485/535 nm in a MicroMax 384 Microwell-Plate Reader (Horiba, Japan). MNPs and MNPs-PEG_12_-AEDP-LD/OmpA were studied at 25 and 50 μg/mL and LPs and MLPs at a 0.1 mg/mL concentration.

### 2.10 MD simulations

#### 2.10.1 OmpA model

A model of the full structure of the OmpA protein was developed with the aid of the X-ray crystallography structures of the transmembrane domain [PDB id. 1QJP (Pautsch and Schulz, 2000) Figure 1A] and the periplasmic domain [PDB id. 2MQE (Ishida et al., 2014) Figure 1A]. These two domains were connected by a disordered linker consisting of 8 amino acids. The 3-dimensional structure of the linker was modeled based on the primary structure available on Uniprot and built using the Pymol^®^ software.

**FIGURE 1:**
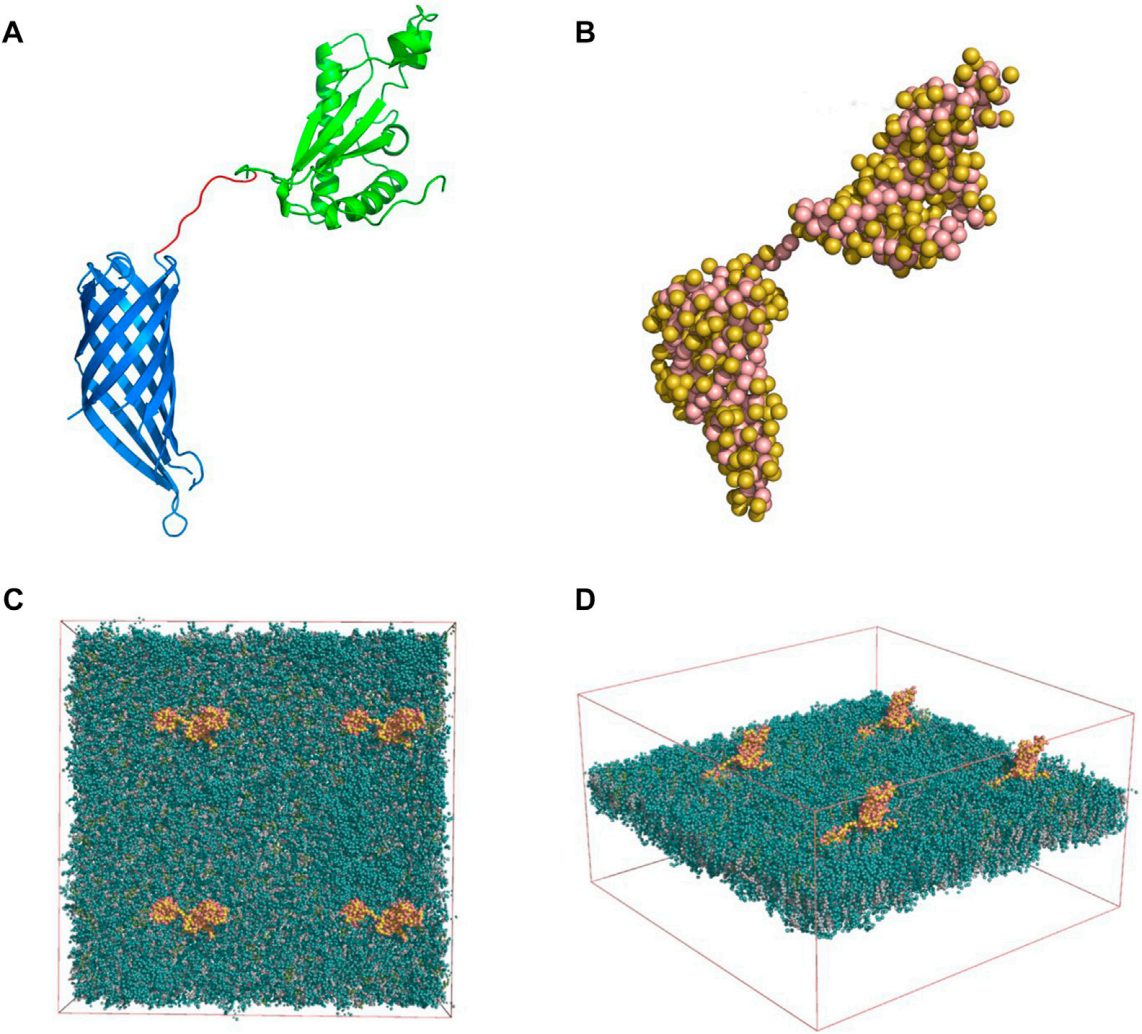
**(A)** Full OmpA structure modeled with the 8 amino-acid linker (red), the periplasmic (green) and transmembrane domains (blue) (Pymol software). **(B)** Martini model of the OmpA protein: Side chains are displayed as yellow beads whereas the backbone beads are shown in pink. Membrane-Protein System: Four OmpA proteins (yellow and pink) inserted in the neuronal membrane (green) top **(C)** and lateral **(D)** views are shown. The solvent beads are not shown for clarity.

Additionally, the structure of the neuronal membrane was obtained from a previously reported work (Ingólfsson et al., 2017). The membrane consists of two (outer and inner) asymmetric leaflets in which their compositions consist of a mixture of Cholesterol, Phosphatidylcholine (PC) Phosphatidylethanolamine (PE) phosphatidylserine (PS) Sphingomyelin (SM) and Glycolipids (Glyco). An equilibrated coarse-grained (CG) conformation of the membrane was solved by explicit MARTINI, which was kindly provided by Helgi Ingolfsson (Ingólfsson et al., 2017) (Livermore National Laboratory, Livermore, United States).

#### 2.10.2 Martini model and elastic network

A coarse-grained (CG) description was adopted throughout this study to enable the simulation at sufficiently large spatial-temporal scales. Here, CG beads represent group of atoms, and their interactions are compressed into a potential interaction energy function (termed force-field). The Martini CG force-field (v 2.2) was used in this study (Monticelli et al., 2008). The atomic model of the OmpA was converted into a CG description by treating each amino acid as a CG bead (depending on their size and chemical properties). This conversion was carried out with the *martinize. py* script (Monticelli et al., 2008). The resulting CG model of the OmpA is shown in Figure 1B.

In order to preserve the secondary and tertiary structure of the OmpA, an elastic network was imposed, separately, on the periplasmic and transmembrane domains. In brief, elastic restraints were applied to all non-neighbor backbone beads within a cut-off distance with upper and lower boundaries of 0.5 and 0.9 nm, respectively (elastic constant of 500 kJ/mol/nm^2^). The elastic network was also generated with the aid of the same *Martinize* script. Note that no elastic network was imposed on the connecting linker.

#### 2.10.3 Membrane-protein system

For the development of the system, 4 identical copies of OmpA were placed in an 2 × 2 arrangement, maintaining their separation almost equal to 20 nm. They were subsequently embedded into the solvated neuronal membrane, yielding a simulation box of size [39.068, 39.068, 20.830] nm (272,095 CG beads). The initial placement of the proteins and their insertion in the membrane were achieved using the GROMACS^®^ package*make index, editconf, genconf* and *solvate* tools (Gromacs user manual version 2018, available in http://manual.gromacs.org/) The resulting simulation system is shown in Figures 1C,D.

#### 2.10.4 MD simulations

MD simulations were carried out with the GROMACS^®^ MD simulation package (version 2018.3) (Van Der Spoel et al., 2005). Sodium and chloride ions were added to the bulk water medium at a concentration of 0.15 M. Electrostatic calculations between beads were considered by a reaction-field approach within a cut-off distance of 1.1 nm. Short-range non-bonded interactions were modeled through a Lennard-Jones potential and only considered within a distance of 1.1 nm. The system was coupled to a V-rescale (Bussi et al., 2007) thermostat to maintain the temperature constant at 310 K (Coupling constant of 1.0 ps). Pressure was also maintained constant at 1 bar by means of a semi-isotropic Parrinello-Rahman (Parrinello and Rahman, 1981) barostat scheme (Coupling constant of 12 ps). Bonded interactions were considered according to the CG Martini model. To avoid large undulations of the bilayer, the head group beads of 62% of the DPPC and 65% of the POPC lipids were weakly position-restrained along the axis orthogonal to the membrane (elastic constant of 2 kJ/mol/nm^2^). Four simulations lasting 2.5 μs were conducted, resulting in a cumulative simulation time of 10 µs, integrating Newton’s equations of motion at discrete time steps of 20 fs. A relaxation of the system of 10 ns preceded the production runs (integration time step of 10 fs). In this relaxation step, the four proteins were kept in their original position (elastic constant of position restraints equal to 1,000 kJ/mol/nm^2^).

### 2.11 Statistical analysis

All results are reported as mean ± standard deviation. Statistical analysis was carried out by employing Graph Pad Prism V 6.01^®^ software (GraphPad Software, United States). Statistical comparisons were performed by using ANOVA followed by a Tukey’s Multiple Comparison test. Results with a *p*-value ≤0.05 (*) were considered statistically different. Symbol * corresponds to statistically significant difference with a *p*-value in the range of 0.01≤ *p*-value ≤0.05, ** to statistically significant difference with a *p*-value in the range of 0.001≤ *p*-value <0.01, *** to *p*-value in the range of 0.0001≤ *p*-value ≤0.00 and **** to *p*-value <0.0001.

## 3 Results and discussion

### 3.1 Synthesis and characterization of MNPs-PEG_12_-AEDP-LD/OmpA, LPs and MLPs

**FIGURE 2:**
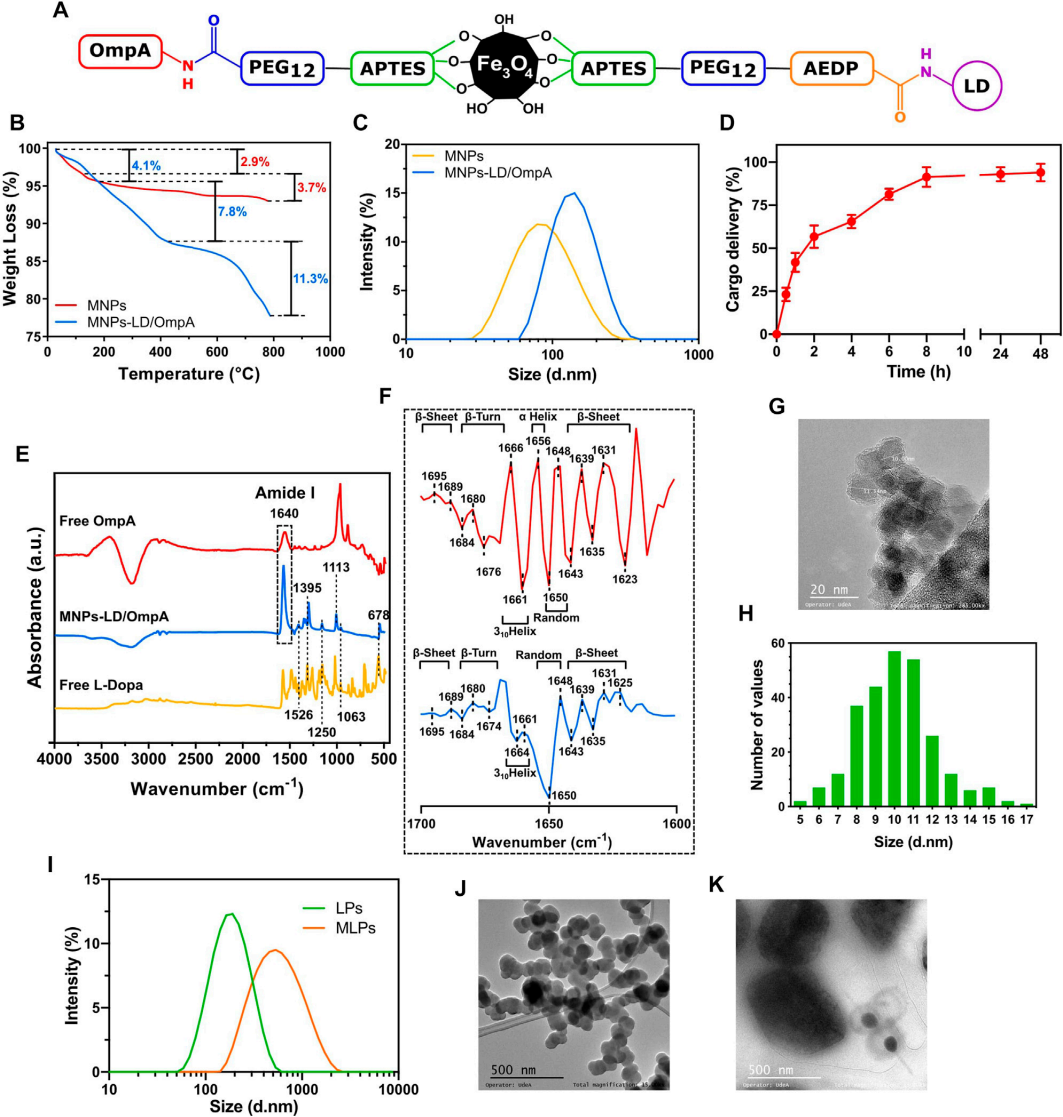
**(A)** Schematic representation of the chemical structure of the developed MNPs-PEG_12_-AEDP-LD/OmpA (Detailed version in Supplementary Scheme S2A). **(B)** TGA thermograms for MNPs and MNPs-PEG_12_-AEDP-LD/OmpA. **(C)** DLS analysis for MNPs and MNPs-PEG_12_-AEDP-LD/OmpA. **(D)** Cargo delivery spectrum for MNPs-PEG_12_-AEDP-LD/OmpA after treatment with DTT. **(E)** FTIR spectra of free LD, free OmpA and MNPs-PEG_12_-AEDP-LD/OmpA. **(F)** Second derivative of the FTIR spectra in the amide I band (1700–1,600 cm^-1^) of free OmpA (red) and immobilized OmpA (MNPs-PEG_12_-AEDP-LD/OmpA, blue). **(G)** TEM image of MNPs-PEG_12_-AEDP-LD/OmpA (scale bar: 20 nm). **(H)** Size distribution histogram of MNPs-PEG_12_-AEDP-LD/OmpA (Based on TEM images). **(I)** DLS analysis for LPs and MLPs. TEM images for LPs **(J)** and MLPs **(K)** (Scale bar: 500 nm).

The chemical structure of the developed MNPs-PEG_12_-AEDP-LD/OmpA nanobioconjugates is shown in Figure 2A (Detailed version on Supplementary Scheme S2A) where it is possible to observe imine and amide bonds formation due to the glutaraldehyde and EDC/NHS crosslinking methods, respectively. In order to verify the correct immobilization of LD and OmpA and the thermal stability of the MNPs-PEG_12_-AEDP-LD/OmpA nanobioconjugates, thermal gravimetric analysis and Fourier transform infrared spectroscopy measurements were performed. Figure 2B shows that the thermograms of MNPs and MNPs-PEG_12_-AEDP-LD/OmpA present a first weight loss of about 2.9% and 4.1% at temperatures ranging from 65°C to 200°C, due to the loss of physically absorbed water on the surface of the NPs. This is following by a second weight loss of 3.7% and 7.8% from 200°C to 400°C, which might be related to residual organic and inorganic compounds adsorbed on the NPs surface (immobilization and synthesis reagents). The MNPs-PEG_12_-AEDP-LD/OmpA thermogram presents a third weight loss of 11.3%, that can be attributed to the detachment of the OmpA protein, LD, AEDP, PEG_12_ and APTES. A step-by-step analysis allows to determine the corresponding weight loss for each molecule. Based on this, 3.1% is related with the functionalization (APTES), 3% with the immobilization of PEG_12_, 2.3% with AEDP, 2% with LD, and 0.9% with the detachment of the OmpA protein. The developed nanobioconjugate shows a high thermal stability below 400°C, that confirms its safeness at physiological temperatures. On the other hand, Figure 2E shows the FTIR spectra of free LD, free OmpA and MNPs-PEG_12_-AEDP-LD/OmpA. The FTIR spectrum of MNPs-LD/OmpA shows multiple peaks associated with characteristic bands of LD at 1,526, 1,395, 1,250, 1,063 and 678 cm^-1^ (Tan et al., 2015). The bands between 1,250 and 678 cm^-1^ can be assigned to C-H aromatic stretching and the peaks at 1,526 and 1,395 cm^-1^ correspond to the aromatic rings (Tan et al., 2015). The peak at 1,113 cm^-1^ is assigned to the Si–O groups, confirming the correct silanization (Yamaura et al., 2004; Can et al., 2009). The strong band at 1,640 cm^-1^ is due to the C=O stretching vibration (Amide I). This corroborates the successful conjugation of the OmpA protein (Lopez-Barbosa et al., 2019).

The size of nanoparticles is one of the most important parameters for the development of successful biomedical applications and particularly in the field of drug delivery due to its close relation with cell viability, magnetic properties, effective cellular uptake, cluster formation, thrombogenesis and prolonged blood circulation times for effective bioavailability (Gupta and Gupta, 2005; Kim et al., 2012; Mou et al., 2015). Due to this, nanobioconjugates size was studied through TEM imaging and DLS measurements. Figure 2C shows the DLS spectra of MNPs and MNPs-PEG_12_-AEDP-LD/OmpA. MNPs showed an average hydrodynamic diameter of about 94.8 ± 42.5 nm while that of the MNPs-PEG_12_-AEDP-LD/OmpA showed an average of about 147.3 ± 51.1 nm. TEM images of MNPs-PEG_12_-AEDP-LD/OmpA showed an average size of 10.1 ± 2.1 nm whereas that of the MNPs present an average of 8.4 ± 1.6 nm. A representative TEM micrograph and the corresponding size distribution histogram of MNPs-PEG_12_-AEDP-LD/OmpA are presented in Figures 2G,H, respectively. Discrepancy between TEM and DLS measurements is attributed to the presence of highly agglomerated clusters of NPs in aqueous solutions. As a result of these clusters, high hydrodynamic diameters are recorded in DLS compared to the individual particle diameters that can be obtained through TEM imaging. Additionally, the difference between the average size of uncoated MNP and the developed MNPs-PEG_12_-AEDP-LD/OmpA nanobioconjugates is attributed to the surface modification (functionalization and immobilization of PEG_12_, AEDP, LD and OmpA). TEM images showed MNPs as dense aggregates due to the lack of any repulsive force between the NPs and the hydrophobic interactions responsible for large clusters (Gupta and Gupta, 2005; Can et al., 2009). Nevertheless, TEM images of MNPs-PEG_12_-AEDP-LD/OmpA showed less aggregates and high dispersity than uncoated MNPs, which could be due to the electrostatic repulsion force and steric hindrance between the OmpA molecules immobilized on the surface of the MNPs (Gupta and Gupta, 2005; Can et al., 2009; Jiang et al., 2014). Previous research has reported a strong relation between the size and the potential body response, which in turn might influence the systemic circulation time of the NPs (Duguet et al., 2006; Shubayev et al., 2009; Kim et al., 2012). According to these studies, NPs with an average diameter above 200 nm are usually sequestered by the reticuloendothelial system (RES) of the spleen and liver *via* mechanical filtration followed by phagocytosis, while those with average diameter below 10 nm are mostly removed through renal clearance (Gupta and Gupta, 2005; Shubayev et al., 2009; Kim et al., 2012). This suggests that based on the average diameter of the manufactured MNPs-PEG_12_-AEDP-LD/OmpA nanobioconjugates are likely to escape renal clearance and RES and therefore, could be administered intravenously as they may have prolonged circulation times and consequently, an increased bioavailability (Gupta and Gupta, 2005; Shubayev et al., 2009). Additionally, diameters of LPs and MLPs were also studied through DLS and TEM. Figure 2I shows the DLS spectra and Figures 2J,K present the TEM images for LPs and MLPs, respectively. LPs showed a hydrodynamic diameter of 208.1 ± 30.2 nm, while that of MLPs was 480.8 ± 25.3 nm. In contrast, their diameters based on TEM images approached 108.6 ± 11 nm and 450 ± 135 nm. These results confirm a significant increase on the LPs’ diameter after NPs uptake. This can be explained by the MLPs synthesis protocol in which mechanical forces are applied to increase interactions between NPs and MLPs, resulting in fusion membrane events and therefore, in larger liposomes. In addition, Supplementary Figure S1 shows Z-potential measurements, time stability and encapsulation efficiency determination for LPs and MLPs. LPs presented an average Z-potential of −30.6 mV, while MLPs −39.9 mV (Supplementary Figure S1A). The obtained surface charges agree well with previously reported values (Leal et al., 2022; Montiel Schneider et al., 2022). These results imply that endocytic pathways are the main internalization routes for these vehicles. Specifically, several studies have suggested macropinocytosis, clathrin-dependent, and caveolin-dependent endocytosis as the most prevalent routes (Rueda-Gensini et al., 2020). Additionally, the average encapsulation efficiency was found to be 72%, and the short-time stability was acceptable (LPs: average size increment of 9.1% and MLPs: 26.8%) after 15 days (Supplementary Figures S1B, S1C). These results are consistent with previous reports, suggesting non-significant effects on cellular biocompatibility and performance (Choi et al., 2019; Ramírez-Acosta et al., 2020; Torres et al., 2022). Recent studies have shown that additional chemical modifications, such as PEGylation can significantly improve the time stability of LP and MLPs (Torres et al., 2022). Therefore, future research should focus on enhancing the performance of the vehicles by increasing the time stability and improving the methods for MLPs synthesis.

### 3.2 Secondary structure analysis of OmpA

The second derivative analysis of the FTIR spectra in the amide I band was carried out to study the potential secondary structural changes of OmpA after the immobilization. This analysis is crucial to determine potential functionality changes associated with alterations in secondary structural features. Figure 2F shows the second derivative amide I spectra for free OmpA (red) and immobilized OmpA (i.e., on the MNPs-PEG_12_-AEDP-LD/OmpA nanobioconjugates, Blue). Free OmpA spectrum showed peaks at 1,695, 1,689, 1,643, 1,639, 1,635, 1,631 and 1,623 cm^-1^, which correspond to β-sheets. Also, at 1,684, 1,680 and 1,676 cm^-1^, which correspond to β-Turns. Additionally, at 1,666 and 1,661 cm^-1^, which correspond to 3_10_Helices. And finally, at 1,656 cm^-1^, which corresponds to α-Helices and 1,650 and 1,648 cm^-1^ that correspond to random coils (Kong and Yu, 2007). These results agree well with previous studies of the OmpA’s secondary structure (Vogel and Jähnig, 1986; Sugawara et al., 1996; Danoff and Fleming, 2011). Because after immobilization, most of the peaks remained at the same wavenumber, no significant structural changes were observed. However, the band at 1,623 shifted to 1,625 cm^-1^, that at 1,676 shifted to 1,674 cm^-1^, the one at 1,666 shifted to 1,664 cm^-1^ and the 1,656 cm^-1^ peak is not present in the OmpA spectrum after immobilization. Further, the presence of a strong peak at 1,650 cm^-1^ suggests an increase in the number of random coils in the structure of the immobilized OmpA. The most relevant secondary structural alterations are related with changes in α-Helical, 3_10_Helical and random coils contents. However, negligible changes were detected in the transmembrane β-barrel domain, suggesting no critical changes in immobilized OmpA functionality with respect to the native state (Lopez-Barbosa et al., 2019).

### 3.3 Nanobioconjugates uptake on liposomes and THP-1 cells

Uptake experiments were carried out to determine the concentration of nanobioconjugates needed for complete saturation of a specific population of LPs and THP-1 cells. Supplementary Figure S5 presents the corresponding results for the uptake analysis on LPs and THP-1 cells. Results clearly show saturation for both LPs and THP-1 cells after treatment with the nanobioconjugates at a concentration of 250 μg/mL. An uptake level above 75% was achieved on THP-1 cells with nanobioconjugates at a concentration of 100 μg/mL, which is the maximum concentration evaluated in this work. Importantly, lower concentrations of nanobioconjugates (below 125 μg/mL) achieved uptake efficiencies of around 20%–60% in cells, whereas those in LPs (at the same concentrations) reached up to 90%. This can be attributed to size and the complexity in the lipid composition of the membranes. Based on these findings, it is possible to conclude that although LPs are highly used models of the cell membrane, its complexity is far to be optimal for the study of internalization dynamics. However, it is a good starting point for rapidly evaluating novel cell-translocating molecules and cell-penetrating nano-systems. Additionally, it is important to highlight that these results confirm association between NPs and liposomes/cells. This association can be related to complete internalization or to interactions between NPs and membranes (NPs could be struck on the surface). Therefore, additional experiments need to be performed in order to elucidate the internalization capacities of NPs (See section 3.7. Cell internalization and endosomal escape analysis).

### 3.4 *In vitro* LD release: redox environment

Quantification of the released LD after exposure to redox environment was determined to corroborate the correct design and functionality of MNPs-PEG_12_-AEDP-LD/OmpA nanobioconjugates. Figure 2D shows the LD delivery percentage after different exposure times to the reducing agent DTT. Release kinetics revealed an initial linear and rapid LD delivery during the first 2 h, reaching a delivery percentage above 50% of the total released LD. After this rapid release, the rate decreased significantly, showing a logarithmic tendency that led to a maximum LD release after 8 h. There were no statistically significant differences in the total cargo delivery percentage after 8, 24 and 48 h. These delivery behavior contrasts with previous works, in which the release rate is lower during the first hours and reaches a maximum after 24 h (Adamo et al., 2017; Hettiarachchi et al., 2021). These results might be explained by the total amount of redox agent employed, suggesting that the higher DTT concentration used, the higher the release rates. In addition, the design and components of vehicles can also impact the release rates of cargos. As a result, the associated steric impediments and spatial distribution of components can generate slower release rates. Finally, results confirmed the correct delivery of LD and therefore, the potential of the developed nanovehicle as a redox-stimuli responsive platform for drug delivery.

### 3.5 Biocompatibility studies and cellular response analysis

**FIGURE 3:**
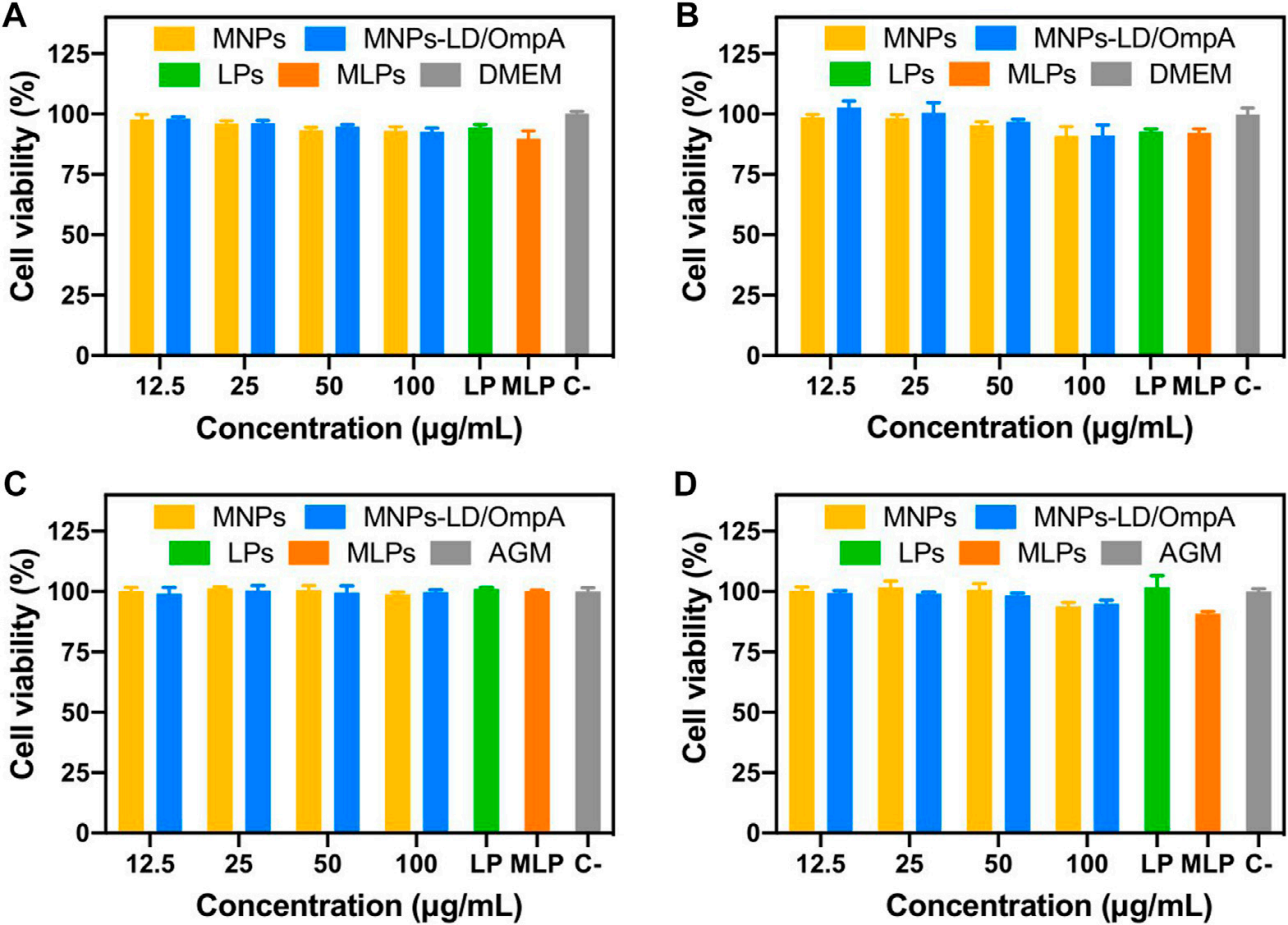
Cytotoxicity assay of MNPs, MNPs-PEG_12_-AEDP-LD/OmpA, LPs and MLPs as tested by LDH assay. Triton X-100 (10% v/v) and DMEM medium were used as positive and negative controls, respectively. SH-SY5Y cells after 24 h **(A)** and 48 h of exposure **(B)**. NHA cells after 24 h **(C)** and 48 h of exposure **(D)**.

To obtain a first insight into the potential of the developed nanovehicle for the treatment of PD as a delivery system for intravenous administration, biocompatibility tests were carried out, including cytocompatibility in T98G, SH-SY5Y, NHA, CP3A4 and MBEC cell lines, hemolysis and platelet aggregation. Figure 3; Supplementary Figure S2 show the cytotoxic effects of MNP, MNPs-PEG_12_-AEDP-LD/OmpA, LPs and MLPs on the different cell lines. Figure 3 shows the results for SH-SY5Y (Figures 3A,B) and NHA cells (Figures 3C,D) after 24 and 48 h of exposure. Additionally, Supplementary Figure S2 presents the results for T98G (Supplementary Figure S2A, S2B), and CP3A4 cells (Supplementary Figure S2C, S2D) after 24 and 48 h of exposure. The results showed high biocompatibility (cell viability above 80%) for MNP, MNPs-PEG_12_-AEDP-LD/OmpA and LPs even after 48 h. These results are easily explained due to the superior biocompatibility reported for the MNPs (Shundo et al., 2012), liposomes (Nguyen et al., 2017), LD (at concentrations below 40 μM) (Lai and Yu, 1997) and OmpA-based nanostructured vehicles (Lopez-Barbosa et al., 2019). Nevertheless, the obtained results for the MLPs showed a marked decrease on the biocompatibility of T98G and NHA cells, and a very subtle one for CP3A4 cells. This can be associated with the enhanced translocation capacity of LPs that led to a significant increase in internalized nanobioconjugates and consequently, in the cytotoxicity. This may be reflected in an increase in bioavailability *in vivo* and therefore in a decrease in the nanobioconjugates dosage required for an optimal therapeutic effect. Importantly, MLPs did not lead to a significant reduction on the viability of SH-SY5Y cells. This can be explained by looking at the physiological functions of astrocytes and their high susceptibility to environmental changes, as has been exemplified for the toxin removal role in neuroprotection mechanisms (Sidoryk-Wegrzynowicz et al., 2011). In this regard, due to the natural ability of astrocytes to remove toxins, it seems to be easier to translocate astrocytes than neurons, which have more highly selective membranes (Chen et al., 2020).

On the other hand, the impact of the nanobioconjugates on the viability and morphology of MBEC cells was also tested. To determine morphological changes, MBEC were evaluated by IFI to assess whether a confluent monolayer was established as measured by the expression of ZO-1 on the plasma membrane (Supplementary Figure S3, top panel). Then, the potential morphological effects of MNPs and MNPs-PEG_12_-AEDP-LD/OmpA on the cell monolayer were evaluated. At 24 h, an accumulation of nanobioconjugates (black spots) was observed on the monolayer at the highest evaluated concentration (50 μg/mL) (Supplementary Figure S3, bottom panel) without affecting the integrity of the cells. On the contrary, when the MBECs were exposed to the highest concentration of LPs (2%), they suffered irreversible morphological damage, evident in the detachment and alteration in the continuity of the monolayer (Supplementary Figure S3, middle panel). Meanwhile, 0.1% of LPs led to no apparent damage at any evaluated time (Supplementary Figure S3).

**FIGURE 4:**
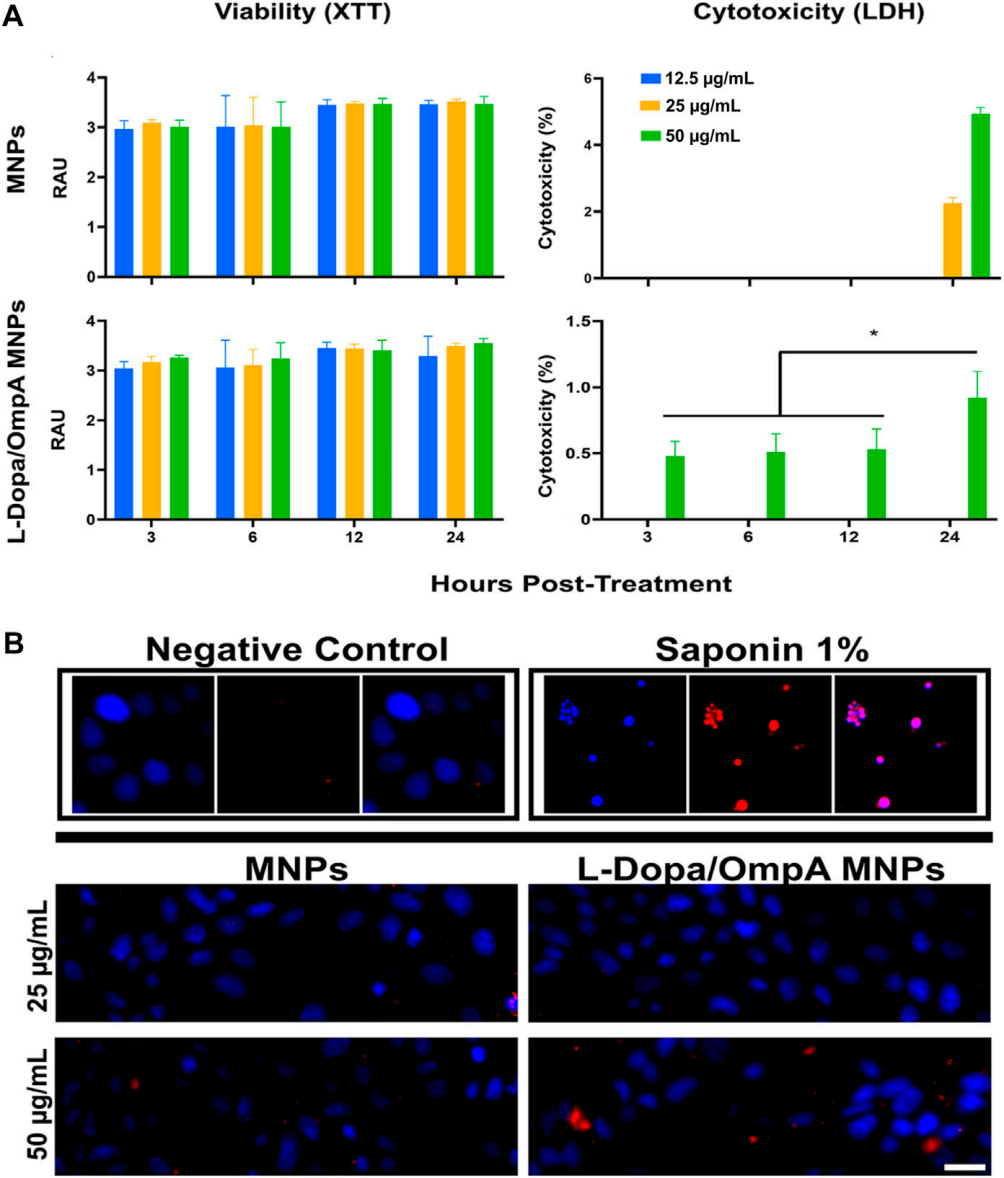
Cell viability and cytotoxicity assays of MNPs, MNPs-PEG_12_-AEDP-LD/OmpA in MBEC cells. **(A)** XTT measurements of cell viability and LDH measurements of cytotoxicity at different exposure times and nanobioconjugates concentrations. **(B)** Fluorescence microscopy images for cell death as assessed by EB and Hoescht nuclear stains (Scale bar: 50 μm). Saponin (1%) was used as a positive control.

These findings were confirmed by evaluating cell viability and cytotoxicity using XTT and LDH release assays, respectively. The XTT assay showed that the viability of cells exposed to MNP or MNPs-PEG_12_-AEDP-LD/OmpA nanobioconjugates remained above 95% for all times and concentrations evaluated. On the contrary, at 12 and 24 h, a non-significant increase in the metabolism of the exposed cells was observed (*p* > 0.05) (Figure 4A). When evaluating cytotoxicity, it was found that at 24 h, the cells treated with bare MNPs at 25 and 50 μg/mL showed an increase of 2% and 6% in the release of LDH, respectively. Interestingly, MNPs-PEG_12_-AEDP-LD/OmpA at 24 h caused a 1% increase in cytotoxicity for cells exposed to the highest nanobioconjugates concentration (50 μg/mL) (*p* < 0.05) (Figure 4A). These results suggest that the nanobioconjugates failed to induce observable metabolic changes or reduce cell survival after the first 24 h. The low cytotoxicity observed was confirmed morphologically using EB and Hoescht nuclear stains (Figure 4B).

Additionally, MNPs-PEG_12_-AEDP-LD/OmpA uptake on MBEC cells was also studied to determine the ability of OmpA to effectively translocate cells without permanent membrane damage. The translocating properties of the nanobioconjugates were evaluated at 37ºC and 4°C to determine the potential energy dependency on the nanobioconjugates internalization mechanism. The obtained results showed that at 4°C, most of the MNPs-PEG_12_-AEDP-LD/OmpA accumulated on the cell surface, and only some managed to come across the cell membrane (Supplementary Figure S4). In contrast, when the cells were incubated at 37°C, the nanoparticles entered and accumulated in close proximity to the nucleus (Supplementary Figure S4). These results suggest that the internalization of MNPs-PEG_12_-AEDP-LD/OmpA takes place by different internalization routes, highlighting energy dependent endocytic pathways as the main route.

**FIGURE 5:**
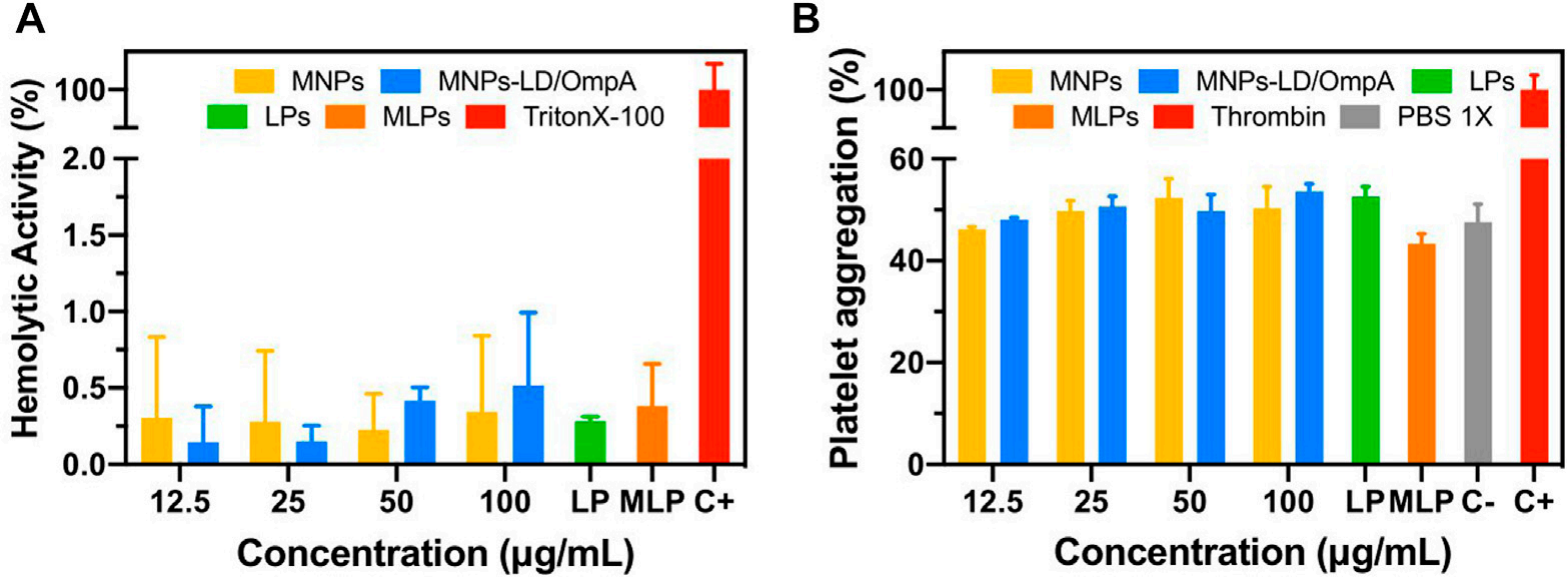
**(A)** Hemolytic effect of MNPs, MNPs-PEG_12_-AEDP-LD/OmpA, LPs and MLPs. Triton X-100 (10% v/v) and PBS 1X were used as positive and negative controls, respectively. **(B)** Platelet aggregation of MNPs, MNPs-PEG_12_-AEDP-LD/OmpA, LPs and MLPs. Thrombin and PBS 1X were used as positive and negative controls, respectively.

Figure 5 shows the hemolytic activity (Figure 5A) and the platelet aggregation effect (Figure 5B) of MNP, MNPs-PEG_12_-AEDP-LD/OmpA, LPs and MLPs. The results demonstrated an average hemolytic activity below 1% for all the treatments. Furthermore, all the treatments showed no significant platelet aggregation compared with the PBS 1X. This suggests no thrombogenic risk and confirms the potential nanovehicle delivery intravenously. According with the ISO standard 10,993: 2009, the obtained results confirm the high hemocompatibility of the developed nanovehicles. These results in conjunction with the high cytocompatibility in a broad variety of cell lines make the developed vehicle a promising drug delivery system for potential applications in the treatment of PD.

### 3.6 FBS stability and protein adsorption assay

Due to the presence of redox agents in blood serum (such as glutathione) as well as thousands of proteins and molecules that can interact and affect the integrity and properties of the nanovehicles, it is crucial to evaluate their stability and performance under conditions that emulate those observed physiologically. Based on this, stability of MNPs-PEG_12_-AEDP-LD/OmpA were determined *via* TGA after exposure to FBS solutions (0%, 10% and 100%) for 7 days (Supplementary Figure S6A). Results showed no statistically significant differences between the weight loss percentages of FBS-exposed MNPs-PEG_12_-AEDP-LD/OmpA with respect to water dispersed MNPs-PEG_12_-AEDP-LD/OmpA. This suggests that after exposure to FBS, MNPs-MNPs-PEG_12_-AEDP-LD/OmpA maintain their integrity and avoid the premature release of LD before reaching the action site.

On the other hand, after the exposure time, MNPs and MNPs-PEG_12_-AEDP-LD/OmpA presented an increase on the weight loss percentage (Supplementary Figure S6B), which can be attributed to serum proteins adsorption. Serum protein deposition plays a fundamental role in the development of drug delivery systems due to the enormous number of proteins present in the blood stream that can completely alter the properties of nanovehicles. This outer layer of serum proteins, commonly called protein corona, directly affect highly relevant factors such as, toxicity, cell uptake, circulation time and clearance, inflammatory responses, organ targeting and biodistribution (Karmali and Simberg, 2011; Chen et al., 2017). The positive or negative effects of this protein corona on the properties of nanovehicles are strongly associated with the type of protein that is adsorbed on the surface. For example, the presence of albumin on protein corona (Desai et al., 2006) as well as surface adsorption of hyaluronan-binding serum proteins (Walkey et al., 2014) has demonstrated a significant increase on cellular uptake for some nanomaterials and targeted cells. Additionally, some have reported that protein corona confers remarkable biocompatibility to highly toxic nanoparticles such as gold and silver (Chen et al., 2017). Moreover, some plasma IgG and complement components such as C1q and C3b (iC3b) had led to reduced circulation time by increasing the uptake on macrophages and therefore, the clarence of NPs (Karmali and Simberg, 2011). In contrast, adsorption of dysopsonins, including albumin and CD47 showed a significant increase in bloodstream circulation time (Chen et al., 2017). In summary, the adsorption of serum proteins might alter the performance of the obtained nanovehicles, and it is therefore crucial to identify the adsorbed proteins and characterize them thoroughly after long-term exposure to whole blood in future work.

### 3.7 Cell internalization and endosomal escape analysis

**FIGURE 6:**
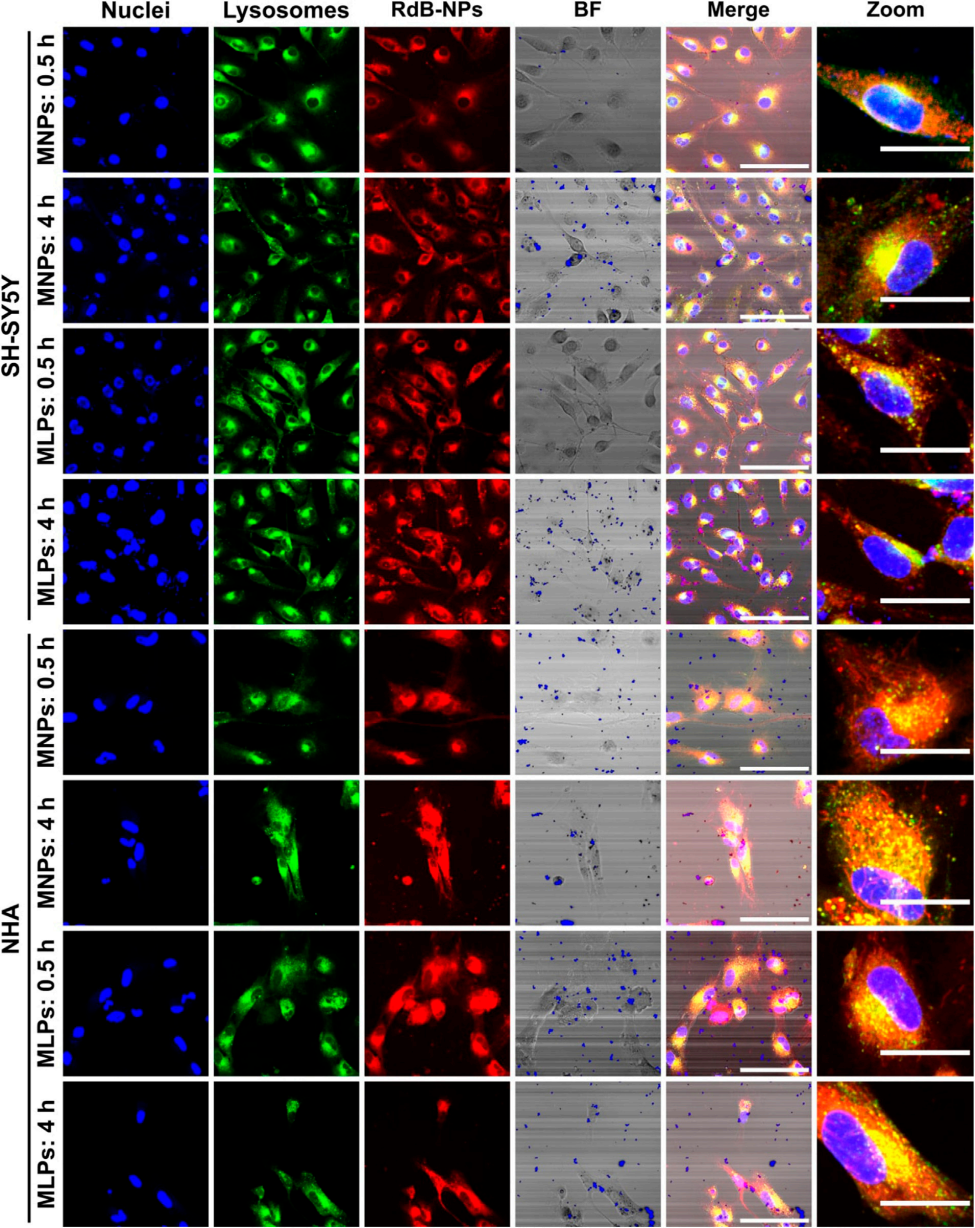
Confocal images for cell internalization and endosomal escape analysis of Rhodamine-B labeled MNPs-PEG_12_-AEDP-LD/OmpA and MLPs in SH-SY5Y and NHA cells at 0.5 and 4 h of exposure. Images were recorded using ×60 magnification and zoom images were obtained by using digital zoom on ×60 magnification images. The scale bars correspond to 100 μm for 60x images and 30 μm for zoom images. In both merge and zoom images, yellow zones point to high colocalization between red and green channels, indicating lysosomal entrapment. In contrast, the presence of non-colocalized red zones suggest endosomal escape.

**FIGURE 7:**
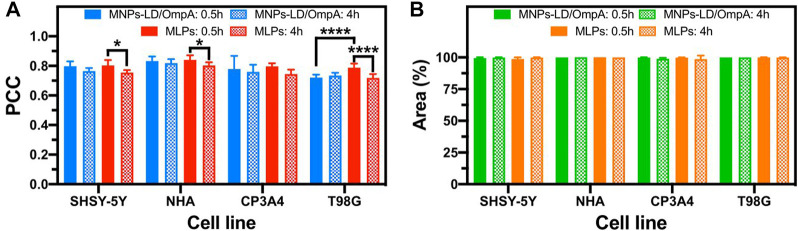
Pearson Correlation Coefficient (PCC) and percentage of intracellular area covered by Rhodamine-B labeled MNPs-PEG_12_-AEDP-LD/OmpA and MLPs after 0.5 and 4 h of exposure in SH-SY5Y, NHA, CP3A4 and T98G cells. **(A)** PCC for MNPs-PEG_12_-AEDP-LD/OmpA and MLPs in different cell lines. **(B)** Covered area percentage for MNPs-PEG_12_-AEDP-LD/OmpA and MLPs in different cell lines. Higher PCC values indicate greater correlation between the red (RhB labeled MNPs-PEG_12_-AEDP-LD/OmpA) and the green (Lysotracker green) channels, suggesting potential lysosomal entrapment. Symbol * corresponds to statistically significant difference with a *p*-value in the range of 0.01≤ *p*-value ≤0.05 and **** to *p*-value <0.0001.

A detailed study of cell internalization and endosomal escape abilities was performed to confirm the translocating potential of MNPs-PEG_12_-AEDP-LD/OmpA and MLPs on different cell lines by using confocal microscopy analysis. Figure 6 and Supplementary Figure S7 show the resulting images for the analysis on SH-SY5Y/NHA cells and T98G/CP3A4 cells, respectively. The presence of a strong red channel signal into the intracellular space confirms the internalization of both MNPs-PEG_12_-AEDP-LD/OmpA and MLPs on the different cell lines. Additionally, the presence of zones of high colocalization (yellow zones in merge images) suggest lysosomal entrapment for a significant portion of internalized nanobioconjugates. These findings support the notion that the internalization process is likely to mainly proceed by the endocytic pathway. However, further studies are needed to gather sufficient information to corroborate this hypothesis (Lopez-Barbosa et al., 2019). Furthermore, Figure 7A presents the quantitative results for colocalization on the different cells lines after 30 min and 4 h of exposure to both treatments. The PCC values for the MNPs-PEG_12_-AEDP-LD/OmpA nanobioconjugates reached 0.797 at 30 min and 0.764 at 4 h in SH-SY5Y, 0.833 and 0.818 in NHA, 0.779 and 0.759 in CP3A4 and 0.720 and 0.734 in T98G cells. These results showed a decrease on the PCC values after 4 h, tendency that can suggest endosomal escape (Smith et al., 2019). Moreover, it can be suggested that the predominant endosomal escape mechanisms are direct translocation and destabilization dynamics as these abilities might be conferred by the OmpA and the polymer, respectively (Lopez-Barbosa et al., 2020; Rueda-Gensini et al., 2020).

On the other hand, MLPs favored significant endosomal escape as well as a notable increase on internalization performance. This can be attributed to additional internalization mechanisms conferred by LPs, namely, membrane fusion and alternative endocytic routes (Miller et al., 1998; Dutta et al., 2011). Membrane fusion contributes to the internalization efficacy by destabilizing the cell membrane through interactions with the positive charges of the choline substructure of soy lecithin, thus allowing the direct release of nanobioconjugates into the cytoplasm (Brooksbank et al., 1993; Dutta et al., 2011).

Previous studies have also reported an increase on endosomal escape mediated by membrane fusion (Rueda-Gensini et al., 2020). This can explain the statistically significant decrease in colocalization after 4 h of exposure for delivery assisted by MLPs. In addition, Figure 7B presents the percentage of covered area for both treatments on the different cell lines at 30 min and 4 h. Results clearly showed percentage of covered areas above 98% for all the tested treatments, confirming the potential of the manufactured nanovehicles as high-performance drug delivery systems. The versatility of these nanovehicles was also evaluated on BBB endothelial cells as a preliminary exploration prior to analysis on *in vitro* models of the BBB. Supplementary Figure S8 shows the results for this cell line, confirming high internalization and an efficient endosomal escape. Finally, low structural damage of cell membranes and absence of nuclei fragmentation and negligible chromatin condensation confirmed the high biocompatibility of the nanobioconjugates (Crowley et al., 2016; Jeon et al., 2019). Although promising results were obtained in the internalization experiments, endosomal escape abilities can be substantially improved. In consequence, future research would be focused on improve the performance by adding different components that can contribute to promoting additional endosomal escape mechanisms. Among these, it is possible to highlight the use of photothermal transduction agents, photosensitizers and fusogenic lipids (Rueda-Gensini et al., 2020).

### 3.8 Parkinson’s disease model: cellular response and antioxidant characterization

**FIGURE 8:**
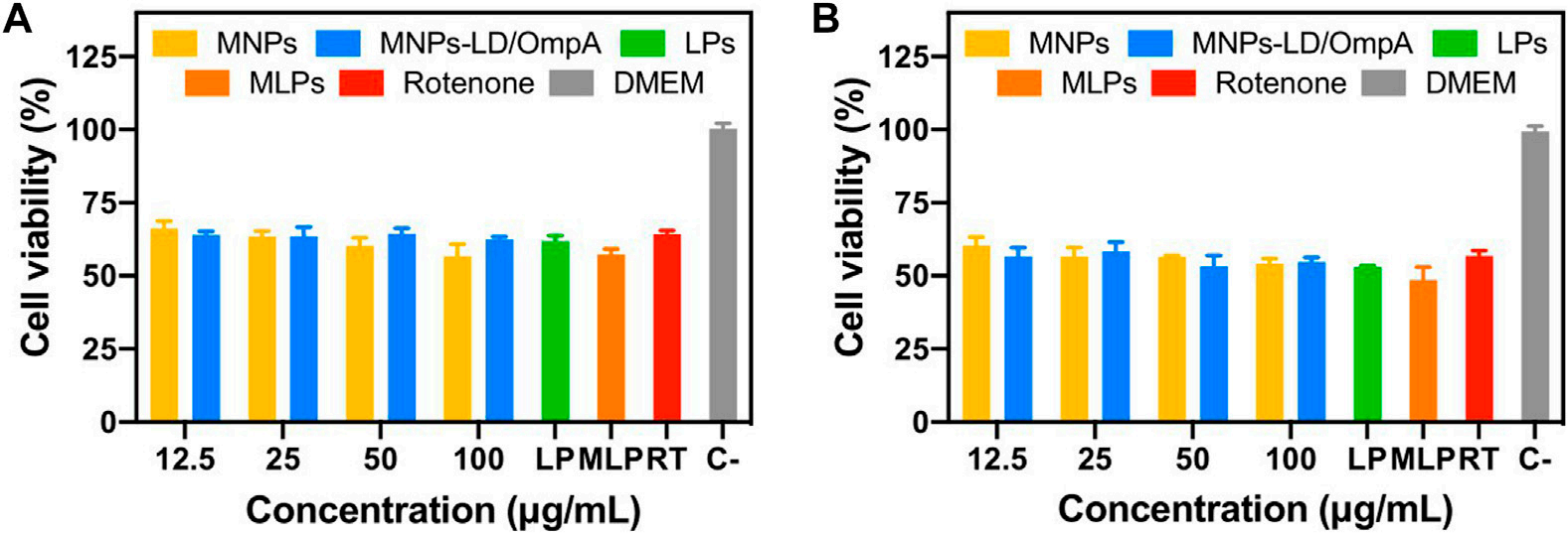
Cytotoxicity assay (LDH) of MNPs, MNPs-PEG_12_-AEDP-LD/OmpA, LPs and MLPs in the SH-SY5Y PD model. PD induced SH-SY5Y cells after 24 h **(A)** and 48 h of exposure **(B)**.

The developed nanovehicles demonstrated high biocompatibility, remarkable internalization rates, and acceptable endosomal escape. However, it is well known that PD drastically impacts cell physiology, potentially changing the response of cells to exogeneous materials. Based on that, an *in vitro* Parkinson’s Disease model was established to test the nanobioconjugates by exposing healthy SH-SY5Y cells to rotenone for simulating the typical pathophysiological mitochondrial dysfunction and oxidative stress of the disease. Figure 8 shows the cytocompatibility results on the PD model for MNPs, MNPs-PEG_12_-AEDP-LD/OmpA, LPs and MLPs. Exposure to rotenone significantly decrease cell viability with respect to healthy cells, which agrees well with previous reports (Cifuentes et al., 2021). Interestingly, none of the treatments negatively impacted cell viability, confirming high biocompatibility, even at highest concentrations. However, previous reports on long-term cytotoxicity for MNPs-based materials have shown negative results (Coccini et al., 2017). Therefore, future work will include long-term cytotoxicity analysis *in vitro* to evaluate the performance of the nanobioconjugates in more challenging and accurate environments.

A preliminary study of the antioxidant potential of the nanobioconjugates was performed *via* DPPH. Supplementary Figure S9 shows the radical scavenging abilities of MNPs and MNPs-PEG_12_-AEDP-LD/OmpA. Results indicate a low activity for MNPs, confirming previously reported works (Shah et al., 2022). In contrast, MNPs-PEG_12_-AEDP-LD/OmpA presented a remarkable antioxidant activity, reaching an inhibition percentage of around 40% at 100 μg/mL. This activity can be attributed to LD, that has been reported as a versatile antioxidant agent (Gülçin, 2007; Colamartino et al., 2015).

**FIGURE 9:**
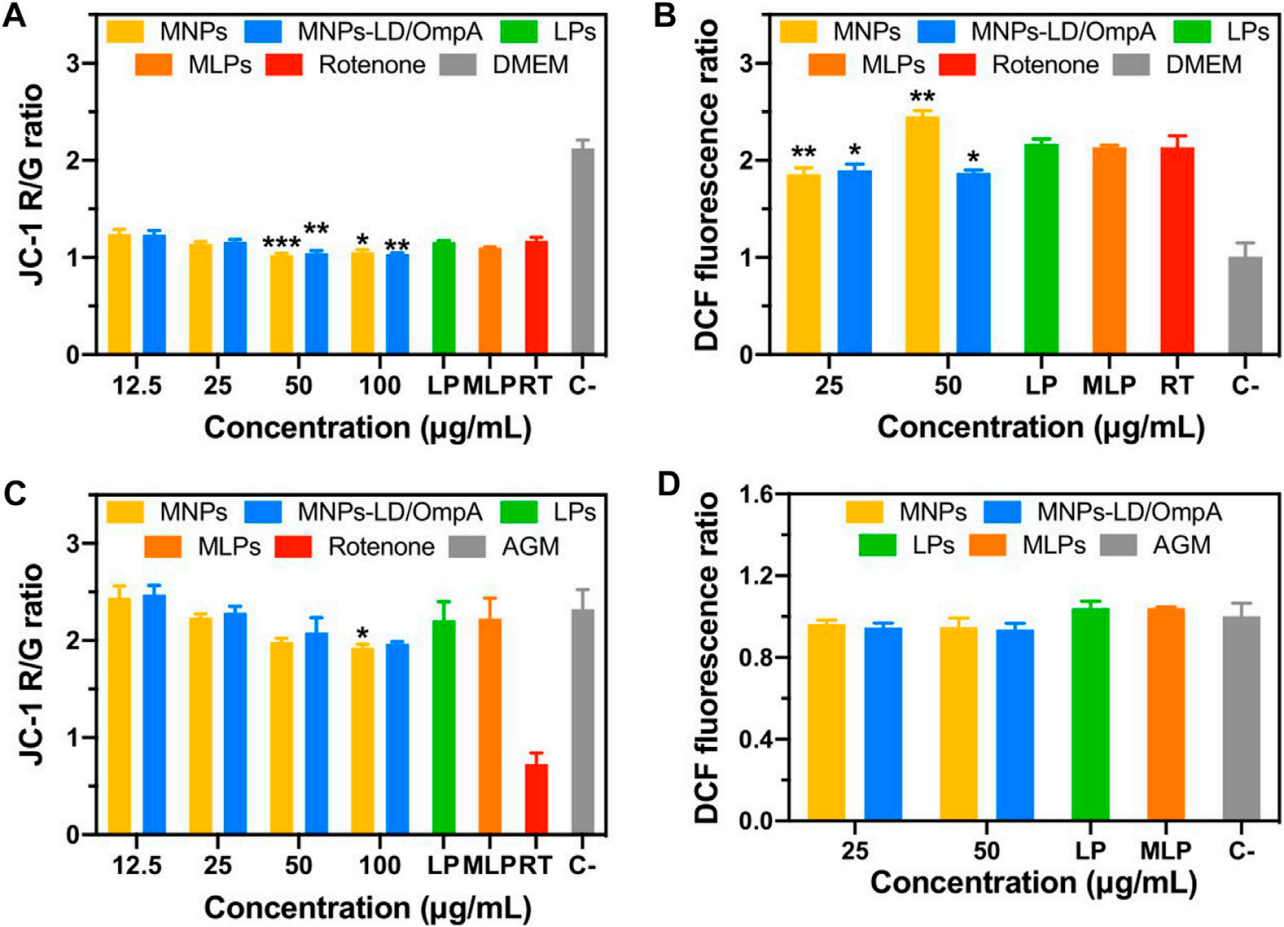
Mitochondrial membrane potential and intracellular ROS for PD induced SH-SY5Y **(A, B)** and NHA cells **(C, D)**. Statistical analysis was performed by comparing the different treatments to the rotenone control for SH-SY5Y cells, and to the AGM medium for NHA cells. Symbol * corresponds to statistically significant difference with a *p*-value in the range of 0.01≤ *p*-value ≤0.05, ** to statistically significant difference with a *p*-value in the range of 0.001≤ *p*-value <0.01 and *** to *p*-value in the range of 0.0001≤ *p*-value ≤0.00.

Additionally, the impact of MNPs, MNPs-PEG_12_-AEDP-LD/OmpA, LPs and MLPs on the mitochondrial function and intracellular ROS generation in the PD model (Figures 9A,B) and NHA cells (Figures 9C,D) was evaluated. The results indicate a negligible impact of MNPs-PEG_12_-AEDP-LD/OmpA on the mitochondrial membrane potential and intracellular ROS generation for both cell lines. In contrast, bare MNPs negatively affect the intracellular ROS generation in the PD model at a concentration of 50 μg/mL. This was not surprising considering previous investigations that have shown a strong correlation between bare MNPs and neurotoxicity through different mechanisms, including iron ions accumulation, mitochondrial dysfunction and oxidative stress (Zhang et al., 2016; Paunovic et al., 2020). Nevertheless, the remarkable performance of MNPs-PEG_12_-AEDP-LD/OmpA and MLPs can be explained by the multifunctional surface functionalization that likely avoids or slows the release of iron ions, thus leading to a minimum impact on iron metabolism (Zhang et al., 2016).

### 3.9 MD simulations

Finally, MD simulations were performed to understand the behavior of OmpA upon interaction with cellular membranes in an attempt to gain a deeper understanding of the mechanisms by which OmpA translocates such membranes. For this, the displacement of the proteins in the *xy*-plane (parallel to the membrane) was calculated for the four OmpA copies. The four protein molecules described random Brownian motion, which corresponds to the lateral diffusion of each molecule independent from each other. As expected, due to the equilibrium conditions, no net positional drift was observed. The mean square lateral displacement ∆X^2^ was also extracted from the simulations. From the slope of the curve (∆X^2^) vs. time, the lateral diffusion coefficient of the protein was estimated to be D = 5.260 μm^2^/s, which is almost 10-fold less than the one found *in vitro* by Ferrage *et al.* for the transmembrane domain (Ferrage et al., 2004).

**FIGURE 10:**
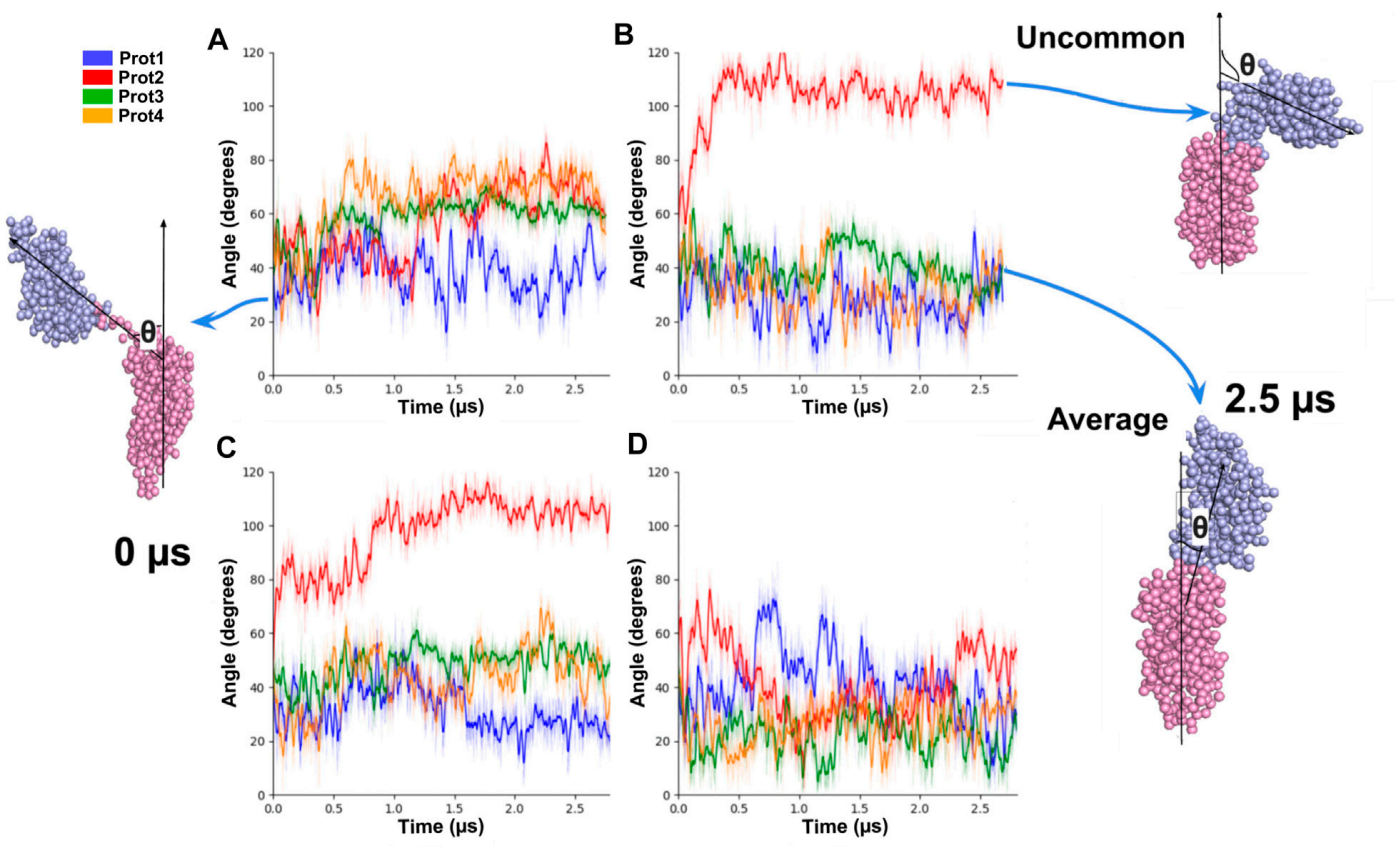
Tilt angles of OmpA molecules for different copies of the protein embedded in the same system. On the left **(A, C)**, the initial conformation of the protein is showed, and its angle relative to the normal of the membrane at 0 μs. On the right **(B, D)**, the conformations of the proteins and their angles at 2.5 μs are shown.

We also examined the tilt angle of the periplasmic domain with respect to the angle normal to the membrane (*z*-axis). In most of the cases (11 out of 16) the tilt angle fluctuated between values from 0º to 60° (Figure 10). In three of the remaining cases, the periplasmic domains adopted an intermediate tilted conformation of 70° (Orange, red, and green curves in Figure 10). For the remaining two cases, two extreme tilted orientations (angles around 100º) were observed (red in Figure 10).

Regarding the localization of phospholipids around OmpA, the considered membrane contains a broad mixture of different types of phospholipids (15 different species). We monitored which of them came in proximity to both the periplasmic and the transmembrane portions of the OmpA. We quantified the time-averaged fraction of phospholipids coming in contact with the protein. From cumulative histograms, we quantified how many of the total number of phospholipids had a fraction in contact with the protein domains (periplasmic in Supplementary Figure S10 or transmembrane in Supplementary Figure S11). Accordingly, an increased localization was manifested in large populations with an elevated fraction in contact. Our analysis revealed a broad pattern of phospholipid-protein interactions in which each group varies its propensity to be in contact with the OmpA, and also depending on which domain of the protein they are binding to. The PODG, a derivative of diacylglycerol and POG3 of glycolipids GM1 showed the highest number of contacts with the periplasmic domain (fraction in contact larger than 0.2 for 60% of the population). Phosphatidylinositol phosphates (PIPs), Phosphatidylinositol (PI) and phosphatidic acid (PA) and phosphatidylserine presented a fair number of contacts with the transmembrane domain of the protein, while they established zero contacts with the periplasmic domain (compare second row of Supplementary Figure S10 with that of Supplementary Figure S11). Furthermore, the POG3 was found to have a greater contact with the periplasmic domain than with the transmembrane domain of the protein (Compare POPG3 of Supplementary Figure S10, S11). Cholesterol (CHOL), which is the more abundant lipid in the membrane by number of molecules, showed almost no interaction with the protein, neither with its transmembrane nor with its periplasmic domain. A similar situation was observed for the molecules belonging to the sphingomyelin and phosphatidylcholine families.

In the case Cerebrosides, one of the groups that is found in higher quantities at the neuronal membrane, interactions with the periplasmic domain were more frequent than with the transmembrane domain (compare middle panel of the fourth row in Supplementary Figure S10, S11). As cerebrosides are not present in the normal lipidic environment of the OmpA, the fact that they have increased interactions with the OmpA periplasmic side suggests that these lipids are likely to provide compensatory interactions for the protein when located in a non-native environment, which is a neuronal membrane in this case.

## 4 Conclusion

Here, we developed a novel multifunctional magnetic and redox-stimuli responsive drug delivery system, based on the combination of soy lecithin liposomes with magnetite nanoparticles functionalized with the high-performance translocating protein OmpA. Throughout this study, we characterized the impact of this vehicle on a broad variety of cell lines, including neuroblastoma, glioblastoma, primary human and rat astrocytes, blood brain barrier rat endothelial cells, primary mice microvascular endothelial cells and PD-induced SH-SY5Y cells. Multifunctional magnetoliposomes demonstrated remarkable biocompatibility in terms of hemocompatibility, platelet aggregation, cytocompatibility and negligible impact on mitochondrial function and intracellular ROS production. Additionally, its versatility for Levodopa delivery, enhanced serum stability, notable cell internalization capacities and acceptable endosomal escape abilities make it a suitable and promising technology for the potential treatment of PD. MD simulations were employed to elucidate the underlying translocating mechanism of OmpA, showing key findings regarding specific interactions with phospholipids that might be useful for the design of next-generation drug delivery nanovehicles based on amphipathic proteins and peptides. Finally, future investigations should focus on characterize the protein corona in detail along with its impact on the performance of the nanovehicle. Moreover, internalization routes must be carefully investigated as well as the stability and functionality of this technology on more complex systemic environments such as animal models and multicellular *in vitro* models. In addition, by incorporating components that strongly improve endosomal escape abilities, such as photothermal transduction agents, photosensitizers and fusogenic lipids, it might be possible to improve the cell-penetrating performance both *in vitro* and *in vivo*.

## Data Availability

The original contribution presented in the study are included in the article/Supplementary Material, further inquiries can be directed to the corresponding author.

## References

[B1] Adamo G. Campora S. Ghersi G. (2017). “Functionalization of nanoparticles in specific targeting and mechanism release,” in Nanostructures for novel therapy (Elsevier), 57–80. 10.1016/B978-0-323-46142-9.00003-7

[B2] Brooksbank D. V. Leaver J. Horne D. S. (1993). Adsorption of milk proteins to phosphatidylglycerol and phosphatidylcholine liposomes. J. Colloid Interface Sci. 161, 38–42. 10.1006/jcis.1993.1437

[B3] Bussi G. Donadio D. Parrinello M. (2007). Canonical sampling through velocity rescaling. J. Chem. Phys. 126, 014101. 10.1063/1.2408420 17212484

[B4] Can K. Ozmen M. Ersoz M. (2009). Immobilization of albumin on aminosilane modified superparamagnetic magnetite nanoparticles and its characterization. Colloids Surfaces B Biointerfaces 71, 154–159. 10.1016/j.colsurfb.2009.01.021 19264459

[B5] Carvalho de Jesus P. da C. Pellosi D. S. Tedesco A. C. (2019). “Magnetic nanoparticles: Applications in biomedical processes as synergic drug-delivery systems,” in Materials for biomedical engineering (Elsevier), 371–396. 10.1016/B978-0-12-816913-1.00012-X

[B6] Chen S.-Y. Tsai S.-T. (2010). The epidemiology of Parkinson’s disease. Tzu Chi Med. J. 22, 73–81. 10.1016/S1016-3190(10)60044-4

[B7] Chen D. Ganesh S. Wang W. Amiji M. (2017). Plasma protein adsorption and biological identity of systemically administered nanoparticles. Nanomedicine 12, 2113–2135. 10.2217/nnm-2017-0178 28805542

[B8] Chen Y. Qin C. Huang J. Tang X. Liu C. Huang K. (2020). The role of astrocytes in oxidative stress of central nervous system: A mixed blessing. Cell Prolif. 53, e12781. 10.1111/cpr.12781 32035016 PMC7106951

[B9] Choi W. I. Sahu A. Wurm F. R. Jo S.-M. (2019). Magnetoliposomes with size controllable insertion of magnetic nanoparticles for efficient targeting of cancer cells. RSC Adv. 9, 15053–15060. 10.1039/c9ra02529d 35516322 PMC9064235

[B10] Cifuentes J. Salazar V. A. Cuellar M. Castellanos M. C. Rodríguez J. Cruz J. C. (2021). Antioxidant and neuroprotective properties of non-centrifugal cane sugar and other sugarcane derivatives in an *in vitro* induced Parkinson’s model. Antioxidants 10, 1040. 10.3390/antiox10071040 34209483 PMC8300827

[B11] Coccini T. Caloni F. Ramírez Cando L. J. De Simone U. (2017). Cytotoxicity and proliferative capacity impairment induced on human brain cell cultures after short- and long-term exposure to magnetite nanoparticles. J. Appl. Toxicol. 37, 361–373. 10.1002/jat.3367 27480414

[B12] Colamartino M. Santoro M. Duranti G. Sabatini S. Ceci R. Testa A. (2015). Evaluation of levodopa and carbidopa antioxidant activity in normal human lymphocytes *in vitro*: Implication for oxidative stress in Parkinson’s disease. Neurotox. Res. 27, 106–117. 10.1007/s12640-014-9495-7 25355370

[B13] Crowley L. C. Marfell B. J. Waterhouse N. J. (2016). Analyzing cell death by nuclear staining with Hoechst 33342. Cold Spring Harb. Protoc. 2016, pdb.prot087205, 10.1101/pdb.prot087205 27587774

[B14] Danoff E. J. Fleming K. G. (2011). The soluble, periplasmic domain of OmpA folds as an independent unit and displays chaperone activity by reducing the self-association propensity of the unfolded OmpA transmembrane β-barrel. Biophys. Chem. 159, 194–204. 10.1016/j.bpc.2011.06.013 21782315 PMC3169180

[B15] Desai N. Trieu V. Yao Z. Louie L. Ci S. Yang A. (2006). Increased antitumor activity, intratumor paclitaxel concentrations, and endothelial cell transport of cremophor-free, albumin-bound paclitaxel, ABI-007, compared with cremophor-based paclitaxel. Clin. Cancer Res. 12, 1317–1324. 10.1158/1078-0432.CCR-05-1634 16489089

[B16] Dong A. Caughey B. Caughey W. S. Bhat K. S. Coe J. E. (1992a). Secondary structure of the pentraxin female protein in water determined by infrared spectroscopy: Effects of calcium and phosphorylcholine. Biochemistry 31, 9364–9370. 10.1021/bi00154a006 1382589

[B17] Dong A. Huang P. Caughey W. S. (1992b). Redox-dependent changes in.beta.-extended chain and turn structures of cytochrome c in water solution determined by second derivative amide I infrared spectra. Biochemistry 31, 182–189. 10.1021/bi00116a027 1310028

[B18] Duguet E. Vasseur S. Mornet S. Devoisselle J.-M. (2006). Magnetic nanoparticles and their applications in medicine. Nanomedicine 1, 157–168. 10.2217/17435889.1.2.157 17716105

[B19] Dutta D. Pulsipher A. Luo W. Mak H. Yousaf M. N. (2011). Engineering cell surfaces via liposome fusion. Bioconjugate Chem. 22, 2423–2433. 10.1021/bc200236m 22054009

[B20] Eriksen J. L. Wszolek Z. Petrucelli L. (2005). Molecular pathogenesis of Parkinson disease. Arch. Neurol. 62, 353. 10.1001/archneur.62.3.353 15767499

[B21] Ferrage F. Zoonens M. Warschawski D. E. Popot J.-L. Bodenhausen G. (2004). Slow diffusion of macromolecular assemblies measured by a new pulsed field gradient NMR method [J. Am. Chem. Soc. 2003, 125, 2541−2545]. J. Am. Chem. Soc. 126, 5654. 10.1021/ja033464g 12603142

[B22] Finsterwald C. Magistretti P. Lengacher S. (2015). Astrocytes: New targets for the treatment of neurodegenerative diseases. CPD 21, 3570–3581. 10.2174/1381612821666150710144502 26166612

[B23] Gülçin İ. (2007). Comparison of *in vitro* antioxidant and antiradical activities of L-tyrosine and L-Dopa. Amino Acids 32, 431–438. 10.1007/s00726-006-0379-x 16932840

[B24] Gupta A. K. Gupta M. (2005). Synthesis and surface engineering of iron oxide nanoparticles for biomedical applications. Biomaterials 26, 3995–4021. 10.1016/j.biomaterials.2004.10.012 15626447

[B25] Haney M. J. Klyachko N. L. Zhao Y. Kabanov A. V. Batrakova E. V. (2018). Extracellular vesicles as drug delivery vehicles for potent redox enzyme catalase to treat Parkinson’s disease. Free Radic. Biol. Med. 128, S18. 10.1016/j.freeradbiomed.2018.10.396

[B26] Hettiarachchi S. D. Cilingir E. K. Maklouf H. Seven E. S. Paudyal S. Vanni S. (2021). pH and redox triggered doxorubicin release from covalently linked carbon dots conjugates. Nanoscale 13, 5507–5518. 10.1039/d0nr08381j 33688879

[B27] Ingólfsson H. I. Carpenter T. S. Bhatia H. Bremer P.-T. Marrink S. J. Lightstone F. C. (2017). Computational lipidomics of the neuronal plasma membrane. Biophysical J. 113, 2271–2280. 10.1016/j.bpj.2017.10.017 PMC5700369 29113676

[B28] Ishida H. Garcia-Herrero A. Vogel H. J. (2014). The periplasmic domain of *Escherichia coli* outer membrane protein A can undergo a localized temperature dependent structural transition. Biochim. Biophys. Acta (BBA) - Biomembr. 1838, 3014–3024. 10.1016/j.bbamem.2014.08.008 25135663

[B29] Jeon H. J. Choi B. B. R. Park K. H. Hwang D. S. Kim U. K. Kim G. C. (2019). Induction of melanoma cell-selective apoptosis using anti-HER2 antibody-conjugated gold nanoparticles. Yonsei Med. J. 60, 509. 10.3349/ymj.2019.60.6.509 31124333 PMC6536400

[B30] Jiang Q. L. Zheng S. W. Hong R. Y. Deng S. M. Guo L. Hu R. L. (2014). Folic acid-conjugated Fe3O4 magnetic nanoparticles for hyperthermia and MRI *in vitro* and *in vivo* . Appl. Surf. Sci. 307, 224–233. 10.1016/j.apsusc.2014.04.018

[B31] Karmali P. P. Simberg D. (2011). Interactions of nanoparticles with plasma proteins: Implication on clearance and toxicity of drug delivery systems. Expert Opin. Drug Deliv. 8, 343–357. 10.1517/17425247.2011.554818 21291354

[B32] Kim J.-E. Shin J.-Y. Cho M.-H. (2012). Magnetic nanoparticles: An update of application for drug delivery and possible toxic effects. Arch. Toxicol. 86, 685–700. 10.1007/s00204-011-0773-3 22076106

[B33] Kong J. Yu S. (2007). Fourier transform infrared spectroscopic analysis of protein secondary structures. Acta Biochim. Biophys. Sin. 39, 549–559. 10.1111/j.1745-7270.2007.00320.x 17687489

[B34] Lai C.-T. Yu P. H. (1997). Dopamine- and l-β-3,4-dihydroxyphenylalanine hydrochloriDe (l-Dopa)-induced cytotoxicity towards catecholaminergic neuroblastoma SH-SY5Y Cells. Biochem. Pharmacol. 53, 363–372. 10.1016/S0006-2952(96)00731-9 9065740

[B35] Leal A. F. Cifuentes J. Quezada V. Benincore-Flórez E. Cruz J. C. Reyes L. H. (2022). CRISPR/nCas9-Based genome editing on GM2 gangliosidoses fibroblasts via non-viral vectors. IJMS 23, 10672. 10.3390/ijms231810672 36142595 PMC9505638

[B36] Lopez-Barbosa N. Suárez-Arnedo A. Cifuentes J. Gonzalez Barrios A. F. Silvera Batista C. A. Osma J. F. (2019). Magnetite–OmpA nanobioconjugates as cell-penetrating vehicles with endosomal escape abilities. ACS Biomaterials Sci. Eng. 6, 415–424. 10.1021/acsbiomaterials.9b01214 33463215

[B37] Lopez-Barbosa N. Garcia J. G. Cifuentes J. Castro L. M. Vargas F. Ostos C. (2020). Multifunctional magnetite nanoparticles to enable delivery of siRNA for the potential treatment of Alzheimer’s. Drug Deliv. 27, 864–875. 10.1080/10717544.2020.1775724 32515999 PMC8216449

[B38] Martin-Jiménez C. A. Gaitán-Vaca D. M. Echeverria V. González J. Barreto G. E. (2017). Relationship between obesity, Alzheimer’s disease, and Parkinson’s disease: An astrocentric view. Mol. Neurobiol. 54, 7096–7115. 10.1007/s12035-016-0193-8 27796748

[B39] Miller C. R. Bondurant B. McLean S. D. McGovern K. A. O’Brien D. F. (1998). Liposome−Cell interactions *in vitro*: Effect of liposome surface charge on the binding and endocytosis of conventional and sterically stabilized liposomes. Biochemistry 37, 12875–12883. 10.1021/bi980096y 9737866

[B40] Monticelli L. Kandasamy S. K. Periole X. Larson R. G. Tieleman D. P. Marrink S.-J. (2008). The MARTINI coarse-grained force field: Extension to proteins. J. Chem. Theory Comput. 4, 819–834. 10.1021/ct700324x 26621095

[B41] Montiel Schneider M. G. Martín M. J. Otarola J. Vakarelska E. Simeonov V. Lassalle V. (2022). Biomedical applications of iron oxide nanoparticles: Current insights progress and perspectives. Pharmaceutics 14, 204. 10.3390/pharmaceutics14010204 35057099 PMC8780449

[B42] Mou X. Ali Z. Li S. He N. (2015). Applications of magnetic nanoparticles in targeted drug delivery system. J. Nanosci. Nanotechnol. 15, 54–62. 10.1166/jnn.2015.9585 26328305

[B43] Nguyen T. L. Nguyen T. H. Nguyen D. H. (2017). Development and *in vitro* evaluation of liposomes using soy lecithin to encapsulate paclitaxel. Int. J. Biomaterials 2017, 1–7. 10.1155/2017/8234712 PMC5346369 28331495

[B44] Niu S. Zhang L.-K. Zhang L. Zhuang S. Zhan X. Chen W.-Y. (2017). Inhibition by multifunctional magnetic nanoparticles loaded with alpha-synuclein RNAi plasmid in a Parkinson’s disease model. Theranostics 7, 344–356. 10.7150/thno.16562 28042339 PMC5197069

[B45] Parrinello M. Rahman A. (1981). Polymorphic transitions in single crystals: A new molecular dynamics method. J. Appl. Phys. 52, 7182–7190. 10.1063/1.328693

[B46] Patil Y. P. Jadhav S. (2014). Novel methods for liposome preparation. Chem. Phys. Lipids 177, 8–18. 10.1016/j.chemphyslip.2013.10.011 24220497

[B47] Paunovic J. Vucevic D. Radosavljevic T. Mandić-Rajčević S. Pantic I. (2020). Iron-based nanoparticles and their potential toxicity: Focus on oxidative stress and apoptosis. Chemico-Biol. Interact. 316, 108935. 10.1016/j.cbi.2019.108935 31870842

[B48] Pautsch A. Schulz G. E. (2000). High-resolution structure of the OmpA membrane domain. J. Mol. Biol. 298, 273–282. 10.1006/jmbi.2000.3671 10764596

[B49] Poewe W. Seppi K. Tanner C. M. Halliday G. M. Brundin P. Volkmann J. (2017). Parkinson disease. Nat. Rev. Dis. Prim. 3, 17013. 10.1038/nrdp.2017.13 28332488

[B50] Ramírez-Acosta C. M. Cifuentes J. Castellanos M. C. Moreno R. J. Muñoz-Camargo C. Cruz J. C. (2020). Ph-responsive, cell-penetrating, core/shell magnetite/silver nanoparticles for the delivery of plasmids: Preparation, characterization, and preliminary *in vitro* evaluation. Pharmaceutics 12, 561. 10.3390/pharmaceutics12060561 32560390 PMC7356180

[B51] Rueda-Gensini L. Cifuentes J. Castellanos M. C. Puentes P. R. Serna J. A. Muñoz-Camargo C. (2020). Tailoring iron oxide nanoparticles for efficient cellular internalization and endosomal escape. Nanomaterials 10, 1816. 10.3390/nano10091816 32932957 PMC7559083

[B52] Schildge S. Bohrer C. Beck K. Schachtrup C. (2013). Isolation and culture of mouse cortical astrocytes. J. Vis. Exp., 50079. 10.3791/50079 23380713 PMC3582677

[B53] Shah S. T. Chowdhury Z. Z. Johan M. R. B. Badruddin I. A. Khaleed H. M. T. Kamangar S. (2022). Surface functionalization of magnetite nanoparticles with multipotent antioxidant as potential magnetic nanoantioxidants and antimicrobial agents. Molecules 27, 789. 10.3390/molecules27030789 35164054 PMC8840749

[B54] Shubayev V. I. Pisanic T. R. Jin S. (2009). Magnetic nanoparticles for theragnostics. Adv. Drug Deliv. Rev. 61, 467–477. 10.1016/j.addr.2009.03.007 19389434 PMC2700776

[B55] Shundo C. Zhang H. Nakanishi T. Osaka T. (2012). Cytotoxicity evaluation of magnetite (Fe3O4) nanoparticles in mouse embryonic stem cells. Colloids Surfaces B Biointerfaces 97, 221–225. 10.1016/j.colsurfb.2012.04.003 22609607

[B56] Sidoryk-Wegrzynowicz M. Wegrzynowicz M. Lee E. Bowman A. B. Aschner M. (2011). Role of astrocytes in brain function and disease. Toxicol. Pathol. 39, 115–123. 10.1177/0192623310385254 21075920 PMC6218934

[B57] Singh A. P. Biswas A. Shukla A. Maiti P. (2019). Targeted therapy in chronic diseases using nanomaterial-based drug delivery vehicles. Signal Transduct. Target. Ther. 4, 33–21. 10.1038/s41392-019-0068-3 31637012 PMC6799838

[B58] Smith S. A. Selby L. I. Johnston A. P. R. Such G. K. (2019). The endosomal escape of nanoparticles: Toward more efficient cellular delivery. Bioconjugate Chem. 30, 263–272. 10.1021/acs.bioconjchem.8b00732 30452233

[B59] Sugawara E. Steiert M. Rouhani S. Nikaido H. (1996). Secondary structure of the outer membrane proteins OmpA of *Escherichia coli* and OprF of *Pseudomonas aeruginosa* . J. Bacteriol. 178, 6067–6069. 10.1128/jb.178.20.6067-6069.1996 8830709 PMC178469

[B60] Tan J. M. Foo J. B. Fakurazi S. Hussein M. Z. (2015). Release behaviour and toxicity evaluation of levodopa from carboxylated single-walled carbon nanotubes. Beilstein J. Nanotechnol. 6, 243–253. 10.3762/bjnano.6.23 25671168 PMC4311623

[B61] Torres C. E. Cifuentes J. Gómez S. C. Quezada V. Giraldo K. A. Puentes P. R. (2022). Microfluidic synthesis and purification of magnetoliposomes for potential applications in the gastrointestinal delivery of difficult-to-transport drugs. Pharmaceutics 14, 315. 10.3390/pharmaceutics14020315 35214047 PMC8877506

[B62] Tysnes O.-B. Storstein A. (2017). Epidemiology of Parkinson’s disease. J. Neural Transm. 124, 901–905. 10.1007/s00702-017-1686-y 28150045

[B63] Van Der Spoel D. Lindahl E. Hess B. Groenhof G. Mark A. E. Berendsen H. J. C. (2005). GROMACS: Fast, flexible, and free. J. Comput. Chem. 26, 1701–1718. 10.1002/jcc.20291 16211538

[B64] Velandia-Romero M. L. Calderón-Peláez M.-A. Castellanos J. E. (2016). *In vitro* infection with Dengue Virus induces changes in the structure and function of the mouse brain Endothelium. PLoS ONE 11, e0157786. 10.1371/journal.pone.0157786 27336851 PMC4919088

[B65] Vogel H. Jähnig F. (1986). Models for the structure of outer-membrane proteins of *Escherichia coli* derived from Raman spectroscopy and prediction methods. J. Mol. Biol. 190, 191–199. 10.1016/0022-2836(86)90292-5 3025450

[B66] Walkey C. D. Olsen J. B. Song F. Liu R. Guo H. Olsen D. W. H. (2014). Protein corona fingerprinting predicts the cellular interaction of gold and silver nanoparticles. ACS Nano 8, 2439–2455. 10.1021/nn406018q 24517450

[B67] Wu Y. Lu Z. Li Y. Yang J. Zhang X. (2020). Surface modification of iron oxide-based magnetic nanoparticles for cerebral theranostics: Application and prospection. Nanomaterials 10, 1441. 10.3390/nano10081441 32722002 PMC7466388

[B68] Yamaura M. Camilo R. L. Sampaio L. C. Macêdo M. A. Nakamura M. Toma H. E. (2004). Preparation and characterization of (3-aminopropyl)triethoxysilane-coated magnetite nanoparticles. J. Magnetism Magnetic Mater. 279, 210–217. 10.1016/j.jmmm.2004.01.094

[B69] Zhang X. Zhang H. Liang X. Zhang J. Tao W. Zhu X. (2016). Iron oxide nanoparticles induce autophagosome accumulation through multiple mechanisms: Lysosome impairment, mitochondrial damage, and ER stress. Mol. Pharm. 13, 2578–2587. 10.1021/acs.molpharmaceut.6b00405 27287467

[B70] Zylberberg C. Gaskill K. Pasley S. Matosevic S. (2017). Engineering liposomal nanoparticles for targeted gene therapy. Gene Ther. 24, 441–452. 10.1038/gt.2017.41 28504657

