# Supplementary material for: Multifunctional magnetoliposomes as drug delivery vehicles for the potential treatment of Parkinson’s disease
Source: Front Bioeng Biotechnol. 2023 May 5;11:1181842. doi: 10.3389/fbioe.2023.1181842 (PMC10196638; doi:10.3389/fbioe.2023.1181842)
Supplement: Supplementary file 1 [file DataSheet1.docx]

Supplementary Material

Multifunctional Magnetoliposomes as Drug Delivery Vehicles for the Potential Treatment of Parkinson’s Disease

Javier Cifuentes, Santiago Cifuentes-Almanza, Paola Ruiz Puentes, Valentina Quezada^1^, Andrés Fernando González Barrios, María-Angélica Calderón-Peláez, Myriam Lucia Velandia-Romero, Marjan Rafat, Carolina Muñoz-Camargo, Sonia L. Albarracín and Juan C. Cruz*

*** Correspondence:** Juan C. Cruz: jc.cruz@uniandes.edu.co

# Supplementary Material and Methods

## Materials

Iron (II) chloride tetrahydrate (98%), iron (III) chloride hexahydrate (97%), acetic acid glacial (99.7%) and sodium hydroxide (NaOH) (98%) were obtained from PanReac AppliChem (Barcelona, Spain). Tetramethylammonium hydroxide (TMAH) (25%), (3-aminopropyl) triethoxysilane (APTES) (98%), N-[3-(dimethylamino)-propyl]-N′-ethylcarbodiimide hydrochloride (EDC) (98%), N-hydroxysuccinimide (NHS) (98%), sodium chloride (NaCl) (99%), glutaraldehyde (25%), amino-PEG12-propionic acid (NH_2_-PEG12-COOH), 3,4-Dihydroxy-L-phenylalanine (Levodopa, L-Dopa, LD) (98%), N,N-Dimethylformamide (DMF) (99.8%), dimethyl sulfoxide (DMSO) (99.9%), isopropylthio-β-galactoside (IPTG) (99%), tryptone, yeast extract, chloramphenicol (98%), soy lecithin (99%), chloroform (99.5%), rhodamine B (95%), phosphate buffered saline (PBS, pH 7.2), Dulbecco’s modified Eagle’s medium F12 (DMEM/F12), 2,2-Diphenyl-1-picrylhydrazyl (DPPH), JC-1, 2,3-Bis(2-methoxy-4-nitro-5-sulfophenyl)-2H-tetrazolium-5-carboxanilide inner salt (XTT) (90%), paraformaldehyde (PFA) (95%), triton X-100 and rotenone (95%) were purchased from Sigma-Aldrich (St. Louis, MO, USA). Fetal bovine serum (FBS), trypsin, and ethylenediaminetetraacetic acid (EDTA) were obtained from BioWest (Riverside, MO, USA). Dulbecco’s modified Eagle’s medium (DMEM), Roswell Park Memorial Institute (RPMI) 1640 medium, ABM Basal Medium, AGM SingleQuots Supplements, Hanks Balanced Salt Solution (HBSS) and penicillin/streptomycin (P/S) were purchased from Lonza (Basel, Switzerland). DCFDA/H2DCFDA-Cellular ROS assay kit was obtained from Abcam (Cambridge, UK). Cytotoxicity Detection Kit (LDH) was purchased from Roche (Basel, Switzerland). Glioblastoma (T98G, ATCC® CRL-1690), neuroblastoma (SH-SY5Y, ATCC® CRL-2266) and THP-1 cells (ATCC TIB-202) were obtained from ATCC (St Cloud, MN, USA). Normal human astrocytes (NHA, Lonza CC-2565) were obtained from Lonza (Basel, Switzerland). Blood Brain Barrier rat endothelial cells (BBB endothelial cells) were provided by Professor Lippmann at Vanderbilt University (USA). 3-[(2-aminoethyl) dithiol] propionic acid (AEDP), LysoTracker Green DND-26, Hoechst 33342 and Ethidium Bromide (EB) were purchased from Thermo Fisher Scientific (Waltham, MA, USA).

## OmpA protein production and purification

### OmpA overexpression in *E. coli*

OmpA overexpression was carried out following the protocol reported by Segura et al. (Segura et al., 2014). Briefly, *E. coli* K-12 w3110/pCA24N OmpA+ was cultured in LB agar plates (NaCl 10 g/L, yeast extract 5 g/L, tryptone 10 g/L and chloramphenicol 50 μg/mL) at 37°C overnight. Then, 50 mL of LB medium supplemented with chloramphenicol were inoculated with an isolated colony and incubated at 37°C and 250 rpm for 24 h. Next, the 50 mL LB medium were added to 500 mL of fresh LB medium and incubated at 37°C and 250 rpm for 24 h. After this, the total volume (550 mL) was added to 4 L of LB medium into a bioreactor (New Brunswick BioFlo/CelliGen 115, New Brunswick Scientific, NJ, USA). The culture was grown at 37°C, 250 rpm and an air flow of 0.2 L/min until reaching an optical density of 0.7 at 600 nm (OD600). Once this point was reached, isopropylthio-β-galactoside (IPTG) (5 g/L) was added to induce OmpA expression. The culture was left in the presence of IPTG for 4 hours.

### OmpA purification and characterization

The bacteria were concentrated by using a tangential filter (Millipore Pellicon-2) (0.22 μm). Then, the concentrated medium was centrifuged (4500 rpm, 4°C) for obtaining a pellet of OmpA overexpressed *E. coli*. For the OmpA extraction, a lysis buffer was added to the bacteria pellet at a ratio of 4 mL per gram of pellet and sonicated for 40 min at 37% amplitude in an ice bath. The resulting solution was then centrifuged (4500 rpm, 4°C) and the supernatant was recovered. OmpA purification was accomplished by immobilized metal affinity chromatography (IMAC), since OmpA was modified with a histidine tail. OmpA presence was verified by SDS-PAGE, which showed a single band at 31 kDa, which corresponded to the molecular weight of OmpA (Segura et al., 2014). Concentration was measured aided by NanoDrop Spectrophotometer (Thermo Fisher Scientific, MA, USA) at 280 nm. Finally, the protein was lyophilized and stored at -20°C until further use.

## MNPs-PEG_12_-AEDP-LD/OmpA, LPs and MLPs characterization

MNPs-PEG_12_-AEDP-LD/OmpA were characterized by Fourier transform infrared spectroscopy (FTIR) and thermogravimetric analysis (TGA) (SDT/Q600, TA Instruments, USA) to confirm effective co-immobilization. Infrared spectra were recorded using a A250 FT-IR (Bruker, Germany). Spectra were recorded for free OmpA, free LD and MNPs-LD/OmpA (4000 - 500 cm^-1^range and 2 cm^-1^spectral resolution). TGA was performed for bare MNPs and for all modified MNPs, which were intermediates in the synthesis of MNPs-PEG_12_-AEDP-LD/OmpA. TGA was carried out by ramping the temperature at a rate of 10°C/min from 25 to 800 °C under a Nitrogen atmosphere (Gas flow of 100 mL/min).

MNPs, MNPs-PEG_12_-AEDP-LD/OmpA, LPs and MLPs sizes were measured by dynamic light scattering technique (DLS) in a Nano ZS zetasizer (Malvern, UK) and by microscopy image analysis in a transmission electron microscope (TEM) Tecnai F30 (FEI Company, USA). Additionally, surface Z-potential was recorded in a Nano ZS zetasizer (Malvern, UK) at room temperature with a solution at pH 7.

In addition, LPs and MLPs short-term stabilities were determined by measuring size changes after 15 days of incubation at 37ºC using a Nano ZS zetasizer (Malvern, UK). Moreover, MLPs encapsulation efficiency was established by analyzing fluorescence changes before and after membrane lysis using Triton X-100. Briefly, 100 μL of MLPs synthesized previously using rhodamine B labeled MNPs-PEG_12_-AEDP-LD/OmpA were seeded into a 96-well microplate for the initial fluorescence measurement. Then, 10 μL of Triton X-100 was added to each well to induce liposomal membrane lysis for the final fluorescence measurement. The membrane rupture leads to the release of the nanobioconjugates, resulting in an increase in the fluorescence intensity. Finally, fluorescence intensity was recorded using a spectrofluorometer (FluoroMax plus C, Horiba, Japan) with excitation and emission wavelengths of 546 nm and 568 nm, respectively. The following equation was employed to calculate the encapsulation efficiency percentage:

$$\text{EE (\%) = 100 * }\frac{\text{(FI (Final) - FI (}\text{Initial}\text{) - FI (}\text{Triton}\text{ X-100))}}{\text{FI (Final)}}$$

Where EE (%) is the encapsulation efficiency percentage, FI (Triton X-100) is fluorescence intensity emission of Triton X-100 (Blank), FI (Initial) is the fluorescence intensity emission before membrane lysis with Triton X-100, and FI (Final) is the fluorescence intensity emission after the treatment with Triton X-100.

## Labeling of MNPs-PEG_12_-AEDP-LD/OmpA with rhodamine B

Rhodamine B (10 mg, 2.1 x 10^-5^mol), EDC (12.3, 6.41 x 10^-5^mol) and NHS (7.4 mg, 6.41 x 10^-5^mol) were dissolved in 2 mL of DMF and diluted in 3 mL of type I water. Then, the rhodamine B solution was heated at 37 °C under continuous magnetic stirring for 15 minutes. This process allows activation of the carboxyl groups of rhodamine B to improve the conjugation with the free amine groups of OmpA in the MNPs-PEG_12_-AEDP-LD/OmpA nanobioconjugates (amide bonds formation). Next, the previously activated rhodamine B solution was added to 40 mL of MNPs-PEG_12_-AEDP-LD/OmpA solution (2.5 mg/mL), sonicated for 5 min (Frequency 40 kHz, amplitude 38%) and left under constant mechanical stirring (200 rpm) for 24 h (protected from light). Finally, rhodamine B labeled MNPs-PEG_12_-AEDP-LD/OmpA were washed 10 times with NaCl solution (1.5% w/v), 5 times with type I water and then, resuspended in 50 mL of type I water, and finally sonicated for 5 min and stored at 4°C until further use.

## Isolation and purification of CP3A4 cells

### Animals

6 males neonatal Wistar rats (*Rattus norvergicus* postnatal days 3-6) were bred in the laboratory of neurobiochemistry of the Science Department at the Pontificia Universidad Javeriana, Bogotá, Colombia. Subjects were hosted in the colony room at a controlled temperature (24±1°C) and light/Dark cycles 12:12 h beginning at 10:00 AM. Animal handling and the methods of euthanize were reviewed and approved by The Institutional Animal Care and Use Committee (CICUAL of Pontificia Universidad Javeriana FUA-066-18).

### Isolation protocol of CP3A4 cells

The protocol described by Schildge et al. (Schildge et al., 2013) was followed with slight modifications. Neonates were euthanized using the physical method of decapitation. Immediately after the decapitation process, brains were removed and located in Hanks Balanced Salt Solution (HBSS) buffer with 1% P/S. Cerebral cortices were carefully removed, and isolated from the rest of the brain. At the end of this procedure, specimens were examined under a stereoscope to assure that they were completely white (clean) without traces of exposed veins or meninges. After the extraction, 300 μL of trypsin 10X was added to the sample and resuspended three times. The obtained mixture was then incubated for 20 min at 37°C and 5% CO_2_ to boost the trypsin. Once the samples were heated, they were resuspended using different volume pipettes (10 mL, 5 mL, and 1 mL glass Pasteur pipettes) until complete homogenization. Immediately after the procedure, 2 mL of supplemented DMEM medium (10% FBS and 1% P/S) were added. Samples were subsequently centrifuged at 1200 rpm for 5 min to collect the cortex tissue. Supernatant was carefully removed from the tube. This procedure was repeated as needed.  Afterwards, 5 mL of DMEM medium was added to the solution and then, samples were resuspended until complete homogenization. Finally, the sample was filtered with a 0.22 μm filter (Corning Falcon™). Cells were seeded at a density of 300.000 cells/mL and incubated under standard conditions. Furthermore, the cells were incubated on a stirring plate at 100 rpm under standard conditions during 24 hours for the purification process. This process was repeated as needed.

## Isolation of MBEC cells

Primary microvascular endothelial cells were isolated from 7-days-old mice (MBEC) following the protocol established by Velandia et al. (Velandia-Romero et al., 2016). Briefly, cerebral cortices were dissociated with DNAse (0.15 ng/mL) and collagenase (1 mg/mL) for 1 h at 37°C. After centrifugation, the cell pellet was placed in a 20% BSA gradient, centrifuged twice at 1000 g for 10 min, and dissociated again with DNAse (0.06 ng/mL), collagenase, and dispase (1 mg/mL) for 50 min at 37°C. Finally, the tissue suspension was centrifuged on a 33% Percoll gradient at 1000 g for 10 min. The resulting pellet (microvessels) was seeded into 12-well plates (coated with collagen type IV and fibronectin -10 μg/mL-) and maintained for three days in DMEM/F12 supplemented with puromycin (3 μg/mL). Finally, the cells were maintained for 20-30 days in DMEM/F12 supplemented with 20% FBS, P/S, 0.7 mM GlutaMAX, 15 U/mL heparin, 1 ng/mL basic fibroblast growth factor (bFGF), and astrocyte conditioned medium.

# Supplementary Schemes and Figures

## Supplementary Schemes


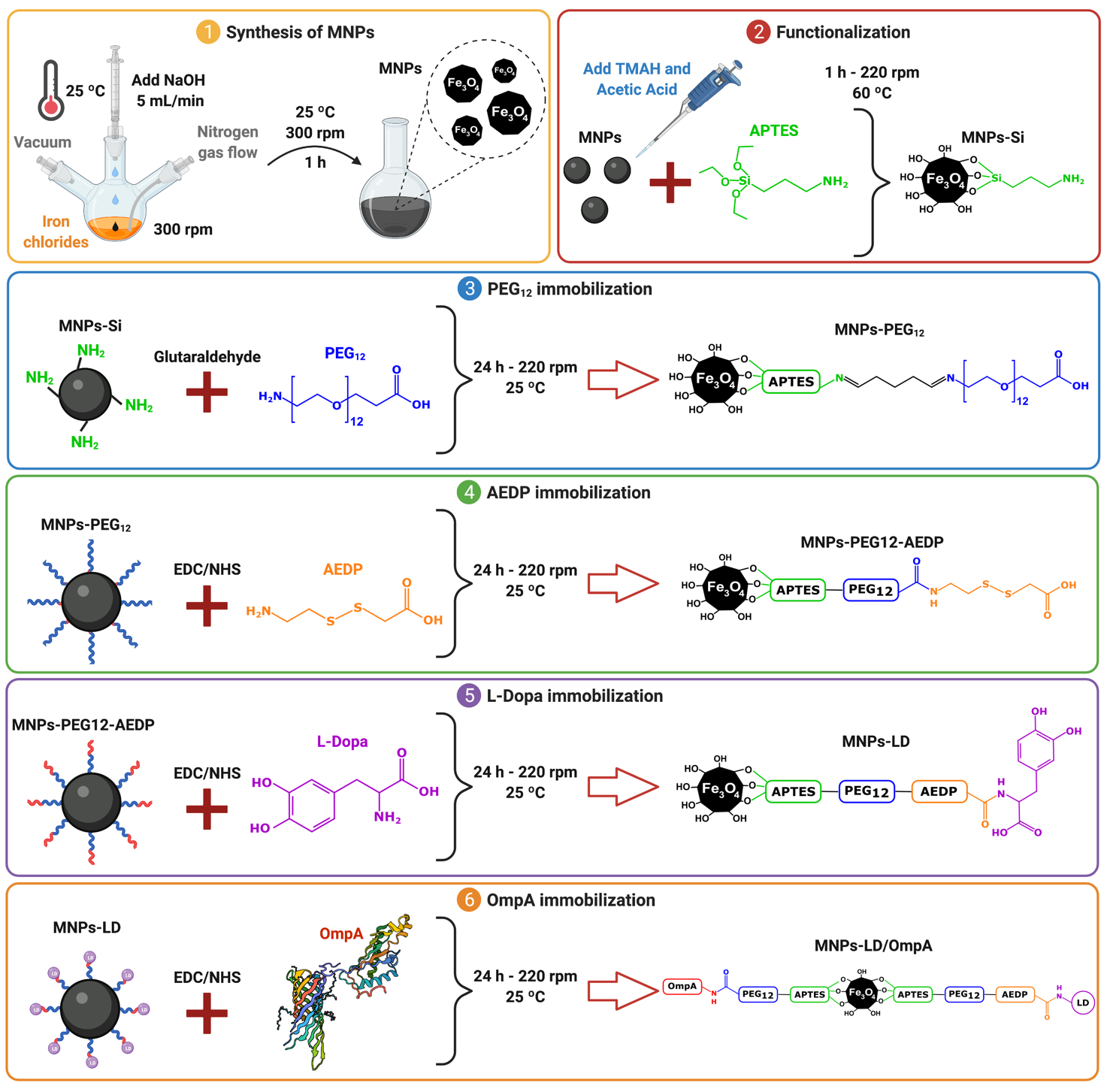


**Supplementary Scheme S1.** Schematic representation of the step-by-step synthesis of MNPs-PEG_12_-AEDP-LD/OmpA and the corresponding chemical structures.


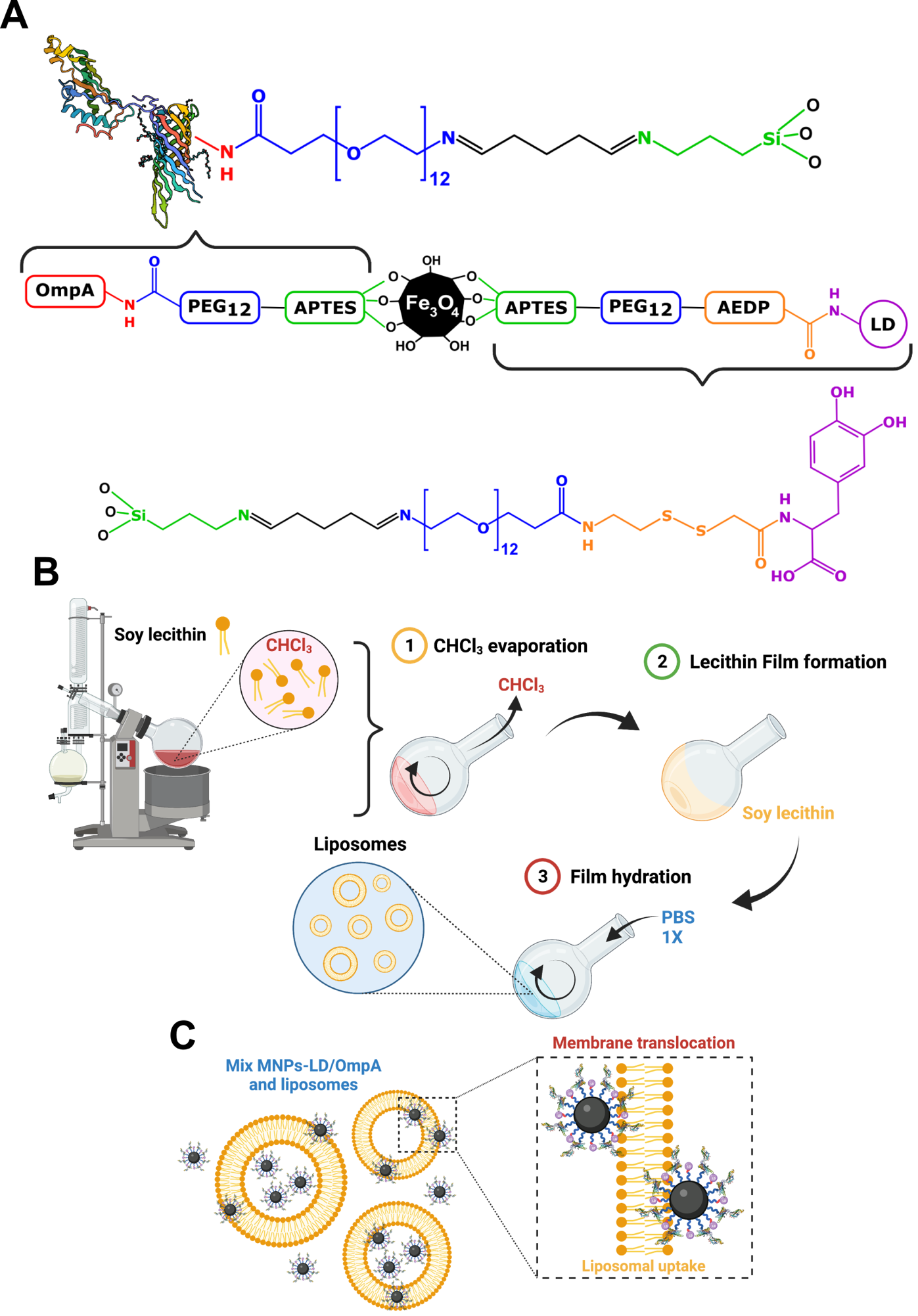


**Supplementary Scheme S2.** Schematic representation of the different components of MLPs including synthesis methods. (**A**) Illustration of the detailed chemical structure of MNPs-PEG_12_-AEDP-LD/OmpA. Illustrations of the synthesis protocols for LPs (**B)** and MLPs (**C**).

## Supplementary Figures

**Supplementary Figure S1.** (**A**) Z-potential of LPs and MLPs. (**B**) MLPs encapsulation efficiency. (**C**) Short-term stability analysis (15 days) for LPs and MLPs.

**Supplementary Figure S2.** Cytotoxicity assay of MNPs, MNPs-PEG_12_-AEDP-LD/OmpA, LPs and MLPs as tested by the LDH assay. Triton X-100 (10% v/v) and DMEM medium were used as positive and negative controls, respectively. T98G cells after 24 h (**A**) and 48 h of exposure (**B**). CP3A4 cells after 24 h (**C**) and 48 h of exposure (**D**).


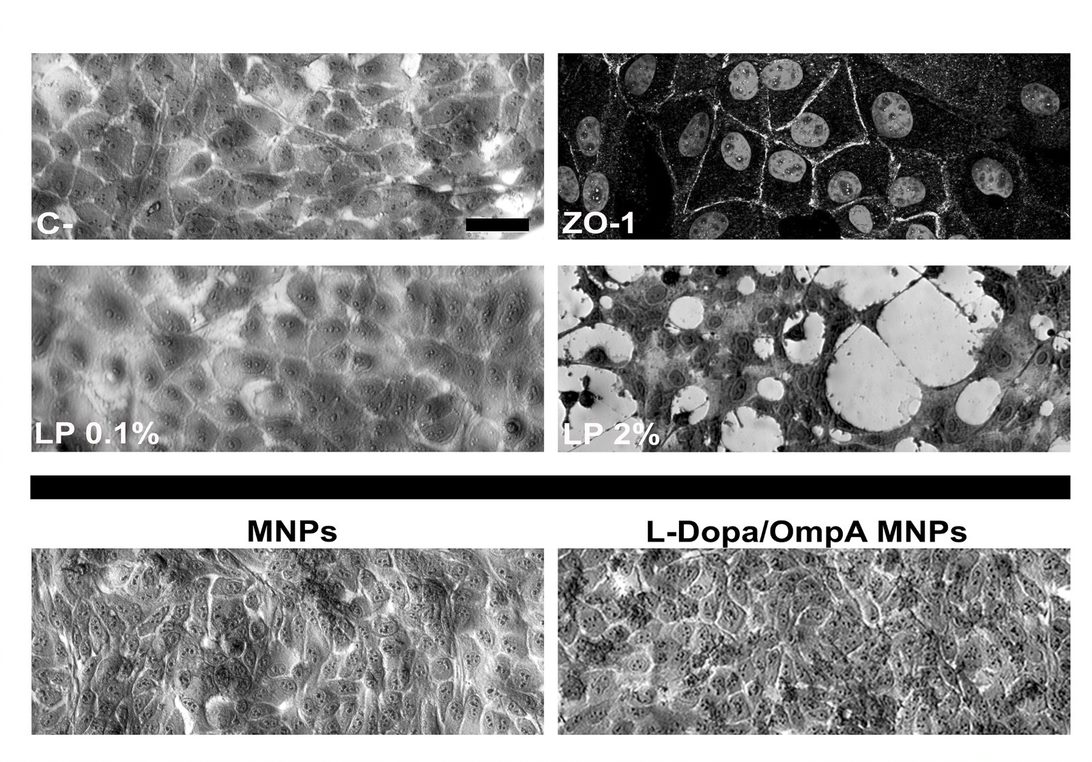


**Supplementary Figure S3.** Evaluation of the MBEC monolayer integrity. Representative image of the monolayer at 24 h of exposure from 3 independent cultures with 2 replicates. The typical morphology of confluent MBEC cells that form a cobblestone pattern (C-) was evaluated by crystal violet staining. On these monolayers, the tight junction protein Zonula Occludens (ZO-1) expression was evaluated by IFI and observed distributed in the membrane delimiting each cell. The damage caused by the positive control after exposure to 2% Liposomes (LP) is shown, where complete destruction of the monolayers was achieved 3 hours post-exposure (hpe). In contrast, the addition of the MNP or MNPs-PEG_12_-AEDP-LD/OmpA nanobioconjugates failed to induce any observable change in cell morphology or affect the integrity of the monolayer.


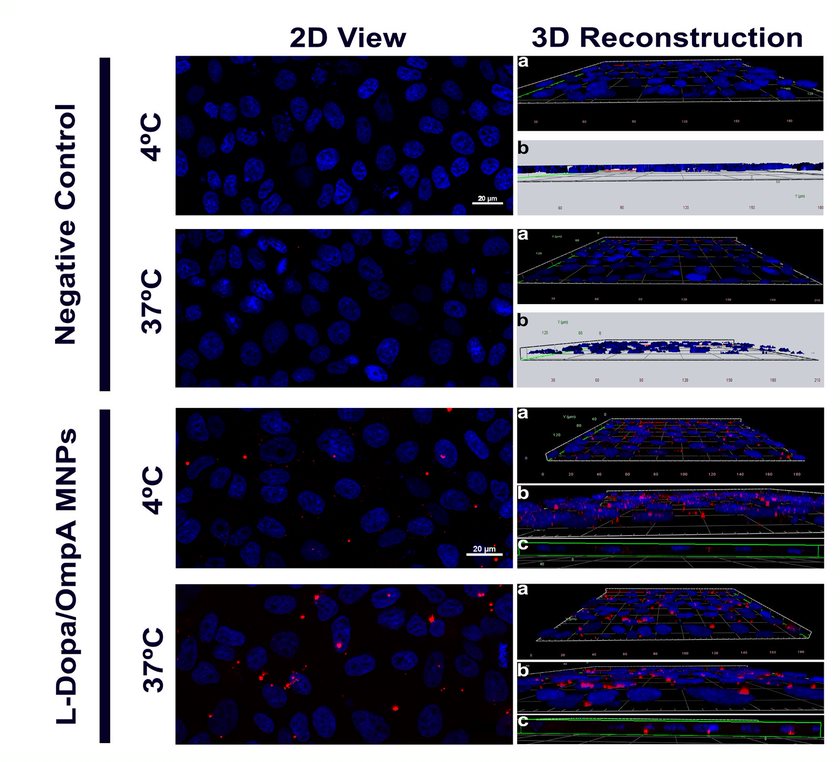


**Supplementary Figure S4.** Fluorescence microscopy images for cellular uptake of MNPs-PEG_12_-AEDP-LD/OmpA in MBEC cells at 4ºC and 37ºC after 75 minutes of exposure. Nuclei were contrasted with Hoechst. 2D images (reconstructed by Apotome using the side view 2D panel tool from the Zen® software) and 3D reconstructions (3D view tool of Zen® software) are presented. Scale bars: 20 μm. (**a**) Top view of the 2D image where the distribution of the nanobioconjugates on the cells is observed. (**b**) Side view show in detail the presence of the nanobioconjugates inside the cells. (**c**) *z*-Stack showing the perinuclear location of the nanobioconjugates.

**Supplementary Figure S5.** Uptake efficiency percentage of MNPs-PEG_12_-AEDP-LD/OmpA on LPs and THP-1 cells. Linear uptake tendency was observed on concentrations below 50 μg/mL. Saturation was achieved at 250 μg/mL for LPs and THP-1 cells.

**Supplementary Figure S6.** Serum stability (**A**) and protein adsorption percentage (**B**) of MNPs-PEG_12_-AEDP-LD/OmpA. Weight loss was determined via TGA. Nanobioconjugates were suspended on type I water, FBS-water solution (10%) and pure FBS (100%) during 7 days at 37ºC under constant stirring. Serum stability was determined by substracting the associated protein adsorption from the total weight loss percentage and comparing it to the type I water thermogram (Weight losses in the temperature range between 400 and 800ºC were plotted). Protein adsorption was calculated as the increase on the weight loss after exposure to FBS proteins.


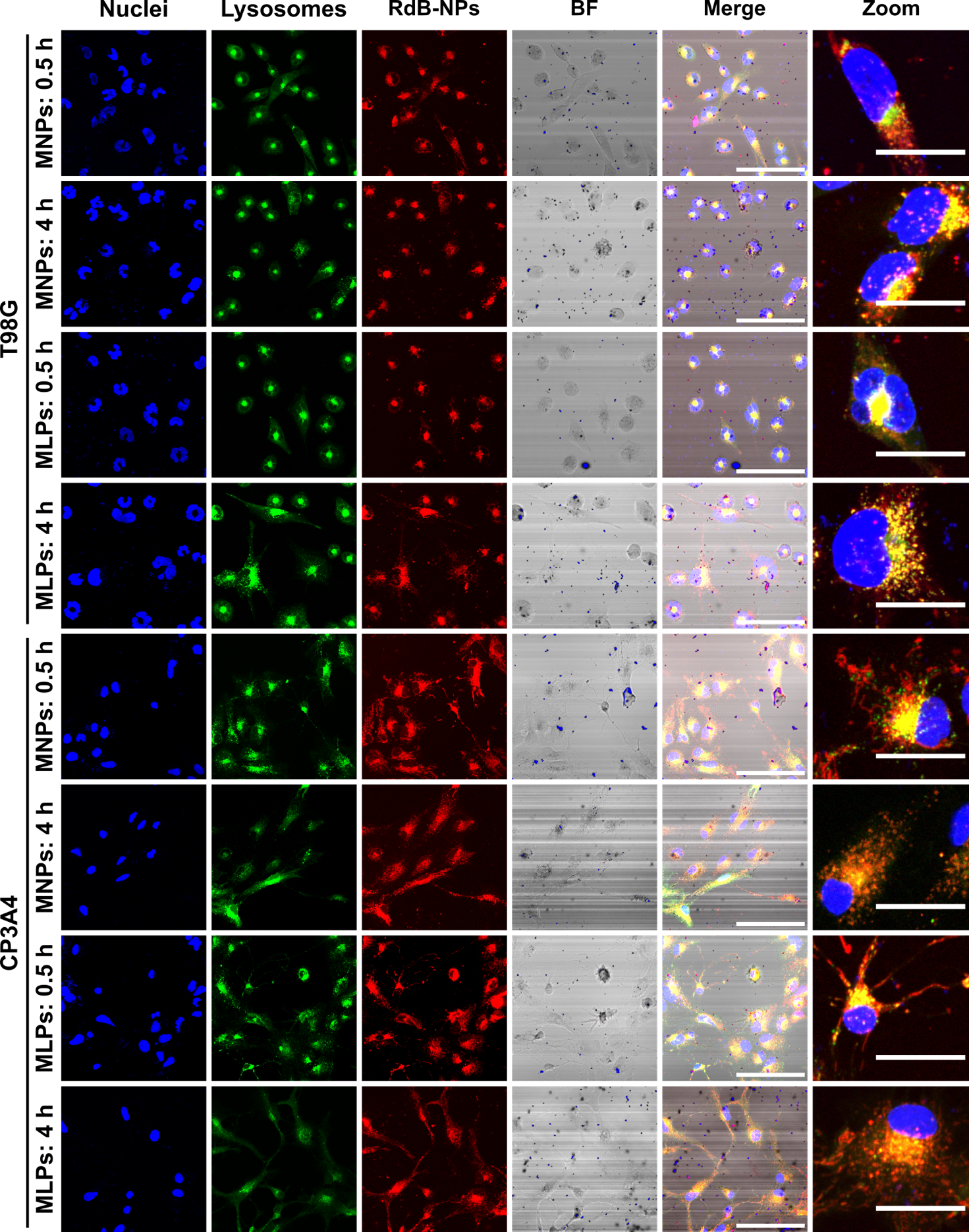


**Supplementary Figure S7.** Confocal images for cell internalization and endosomal escape analysis of Rhodamine-B labeled MNPs-PEG_12_-AEDP-LD/OmpA and MLPs in T98G and CP3A4 cells at 0.5 and 4 h of exposure. Images were recorded using 60x magnification and zoom images were obtained by using digital zoom on 60x magnification images. The scale bars correspond to 100 μm for 60x images and 30 μm for zoom images. In both merge and zoom images, yellow zones point to high colocalization between red and green channels, indicating lysosomal entrapment. In contrast, the presence of non-colocalized red zones suggest endosomal escape.


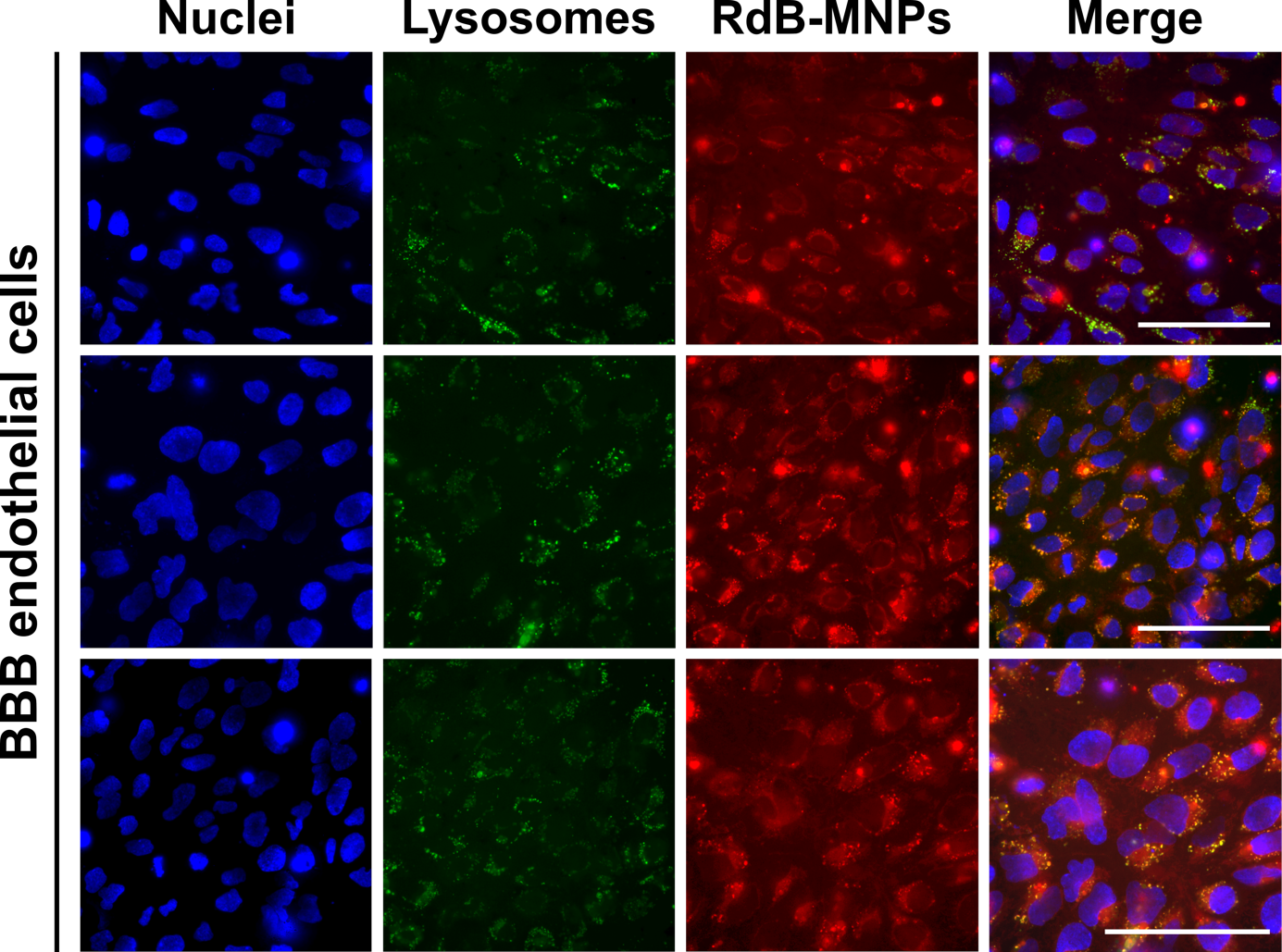


**Supplementary Figure S8.** Fluorescence microscopy images for cell internalization and endosomal escape analysis of Rhodamine-B labeled MNPs-PEG_12_-AEDP-LD/OmpA in BBB endothelial cells at 0.5 h of exposure. Images were recorded using 40x magnification and zoom images (third row) were obtained by using digital zoom on 40x magnification images. The scale bars correspond to 100 μm. In the merged images, yellow zones point to high colocalization between red and green channels, indicating lysosomal entrapment. In contrast, the presence of non-colocalized red zones suggest endosomal escape.

**Supplementary Figure S9.** Antioxidant capacities of MNPs and MNPs-PEG_12_-AEDP-LD/OmpA as measured by the DPPH analysis. Free LD was used as a control.

**Supplementary Figure S10.** Cumulative histograms of the average number of contacts between phospholipids and the periplasmic domain of the OmpA. Each panel shows the histogram for a family of phospholipids. First row: Phospatidylcholine (PC), Phosphatidylethanolamine (PE), Sphingomyelin (SM). Second row: Phosphatydilinositol Phosphates (PIPs), Phosphatidylinositol (PI) and Phosphatidic acid (PA), Phosphatidylserine (PS). Third row: Diacylglycerol (DAG) and Cholesterol (CHOL), Lysophosphatidylcholine (LPC) and Lysophosphatidylethanolamine (LPE), Glycolipid (GM3). Fourth row: Glycolipids (GM1), Cerebrosides, Ceramides (CER).

**Supplementary Figure S11.** Cumulative histograms of the average number of contacts with the transmembrane domain of the OmpA. Each panel shows the histograms for a family of phospholipids. First row: Phospatidylcholine (PC), Phosphatidylethanolamine (PE), Sphingomyelin (SM). Second row: Phosphatydilinositol Phosphates (PIPs), Phosphatidylinositol (PI) and Phosphatidic acid (PA), Phosphatidylserine (PS). Third row: Diacylglycerol (DAG) and Cholesterol (CHOL), Lysophosphatidylcholine (LPC) and Lysophosphatidylethanolamine (LPE), Glycolipid (GM3). Fourth row: Glycolipids (GM1), Cerebrosides, Ceramides (CER).

# Supplementary References

Schildge, S., Bohrer, C., Beck, K., and Schachtrup, C. (2013). Isolation and Culture of Mouse Cortical Astrocytes. *JoVE*, 50079. doi: 10.3791/50079.

Segura, S. M. A., Macías, A. P., Pinto, D. C., Vargas, W. L., Vives-Florez, M. J., Barrera, H. E. C., et al. (2014). “Escherichia coli’s OmpA as Biosurfactant for Cosmetic Industry: Stability Analysis and Experimental Validation Based on Molecular Simulations,” in *Advances in Computational Biology* Advances in Intelligent Systems and Computing., eds. L. F. Castillo, M. Cristancho, G. Isaza, A. Pinzón, and J. M. C. Rodríguez (Cham: Springer International Publishing), 265–271. doi: 10.1007/978-3-319-01568-2_38.

Velandia-Romero, M. L., Calderón-Peláez, M.-A., and Castellanos, J. E. (2016). In Vitro Infection with Dengue Virus Induces Changes in the Structure and Function of the Mouse Brain Endothelium. *PLoS ONE* 11, e0157786. doi: 10.1371/journal.pone.0157786.
